# PPARα−ACOT12 axis is responsible for maintaining cartilage homeostasis through modulating de novo lipogenesis

**DOI:** 10.1038/s41467-021-27738-y

**Published:** 2022-01-05

**Authors:** Sujeong Park, In-Jeoung Baek, Ji Hyun Ryu, Churl-Hong Chun, Eun-Jung Jin

**Affiliations:** 1 Department of Biological Sciences, College of Natural Sciences, Wonkwang University, Iksan, Chunbuk 54538 Korea; 2 Asan Institute for Life Sciences, University of Ulsan College of Medicine, Seoul, 05505 Republic of Korea; 3 Department of Carbon Convergence Engineering, Wonkwang University, Iksan, Chunbuk 54538 Korea; 4 Department of Orthopedic Surgery, Wonkwang University School of Medicine, Iksan, Chunbuk 54538 Korea

**Keywords:** Extracellular matrix, Osteoarthritis, Chondrocytes

## Abstract

Here, in *Ppara*^*−/−*^ mice, we found that an increased DNL stimulated the cartilage degradation and identified ACOT12 as a key regulatory factor. Suppressed level of ACOT12 was observed in cartilages of OA patient and OA-induced animal. To determine the role and association of ACOT12 in the OA pathogenesis, we generated *Acot12* knockout (KO) (*Acot12*^*−/−*^) mice using RNA-guided endonuclease. *Acot12*^*−/−*^ mice displayed the severe cartilage degradation with the stimulation of matrix MMPs and chondrocyte apoptosis through the accumulation of acetyl CoA. Delivery of acetyl CoA-conjugated chitosan complex into cartilage stimulated DNL and cartilage degradation. Moreover, restoration of ACOT12 into human OA chondrocytes and OA-induced mouse cartilage effectively rescued the pathophysiological features of OA by regulating DNL. Taken together, our study suggested ACOT12 as a novel regulatory factor in maintaining cartilage homeostasis and targeting ACOT12 could contribute to developing a new therapeutic strategy for OA.

## Abstract (web summary)

Increasing evidence suggested that dysregulation in lipid metabolism is linked to OA pathogenesis, but the underlying regulatory mechanism is not well understood. Here, the authors show that PPARα-ACOT12 signalling regulates cartilage homeostasis by regulating de novo lipogenesis in mice.

## Introduction

Chondrocytes embedded within the extracellular matrix maintain cartilage homeostasis by stimulating anabolic and catabolic pathways to regulate production, turnover, and degradation of cartilage matrix proteins, such as type II collagen and proteoglycan^1^. During the pathogenesis of osteoarthritis (OA), chondrocytes produce extracellular matrix-degrading proteins such as matrix metalloproteinases (MMPs) as well as pro-inflammatory cytokines such as interleukin (IL)-1 and tumor necrosis factor (TNF) leading to the loss of chondrocyte cellularity and cartilage degeneration^2–6^. OA is a complex disease complicated with several metabolic diseases including dyslipidemia, hyperglycemia, and hypertension^7–9^. Epidemiological studies suggested that obese individuals have significantly increased the incidence of OA^10–12^. Increased levels of total free fatty acid (FFA) have been reported in OA patients^13–15^ and prior to the appearance of histopathological OA features, accumulation of substantial LD in articular cartilage has been reported^9,16,17^. The elevated level of FFA in chondrocytes may contribute to the accumulation of LDs in articular cartilage^18^. Increased level of saturated fatty acids (SFAs) is closely related to the progression of cartilage degradation^19,20^. High susceptibility to SFA-rich diet is associated with increased OA-like pathophysiological changes^20,21^ and SFA deposits in the cartilage alter the cartilage metabolism to be more prone to damage^20^. Recently, our laboratory also reported that regardless of body mass index (BMI), abnormal lipid accumulation in OA chondrocytes may responsible for the development and progression of OA^22^. Moreover, regardless of obesity, an increased level of serum cholesterol has been reported to be closely associated with the OA progression^14,23^. Although there is an increasing evidence that excessive lipid deposition (LD) could be associated with OA pathogenesis, the functional role and regulatory mechanism of LD during OA pathogenesis has not been well established. Therefore, understanding how LDs are regulated and accumulated in chondrocytes and how LD affects the pathogenesis of OA could have a great impact on developing a novel approach or techniques for treating OA.

De novo lipogenesis (DNL) and fatty acid (FA) oxidation, which regulates the breakdown and synthesis of fatty acids, are important determinants of lipid accumulation^24,25^. Lipogenesis is the process that synthesizes the FA chain by combining the acetyl group which adds carbons to a growing FA chain^26^. DNL is a highly regulated pathway and begins with acetyl-CoA as a principal building block for de novo synthesis of fatty acids^26,27^. Acetyl-CoA is a key metabolic intermediate in DNL. Acetyl-CoA, a key indicator of the metabolic state is regulated by adenosine triphosphate (ATP)-citrate lyase (ACLY), acetyl-CoA synthetase 1 (ACSS1), acetyl-CoA synthetase 2 (ACSS2), and acyl-CoA thioesterases (ACOTs). ACLY, ACSS1, and ACSS2 are involved in the production of acetyl-CoA and ACOTs hydrolyze fatty acyl-CoA into free FA and CoA, contributing to pyruvate metabolism and peroxisomal and mitochondrial fatty acyl-CoA oxidation^28–32^. Among them, *ACOT12*, also known as StarD15 or cytosolic acetyl-CoA hydrolase, is the major cytoplasmic enzyme that preferentially hydrolyzes the thioester bond of acetyl-CoA and generates acetate and CoA^27,33^. Since ACOT12 may responsible for determining the rate of degradation of cytosolic acetyl-CoA by controlling their dispositions towards oxidation versus complex lipid synthesis, the elucidation of the biological role and function of ACOT12 could provide a molecular basis and mechanism for deregulation of lipid homeostasis involved in the OA pathogenesis and could contribute in developing a new therapeutic approach for OA pathogenesis. In this study, we found that lipid accumulation increased by PPARα deficiency stimulates cartilage degradation through the modulation of ACOT12. In addition, the significant suppression of *ACOT12* in the cartilage of human OA patient and OA animal models, indicating a requisite role of ACOT12 in maintaining articular cartilage homeostasis. We have also demonstrated that *ACOT12* deficiency is involved in the LD accumulation of the stimulation of de novo lipogenesis (DNL) through the accumulation of acetyl-CoA, leading to the stimulation of cartilage-degrading enzymes and apoptosis of chondrocyte.

## Results

### PPARα is responsible for the accumulation of obese-independent LD in the OA articular chondrocytes

**Fig. 1:**
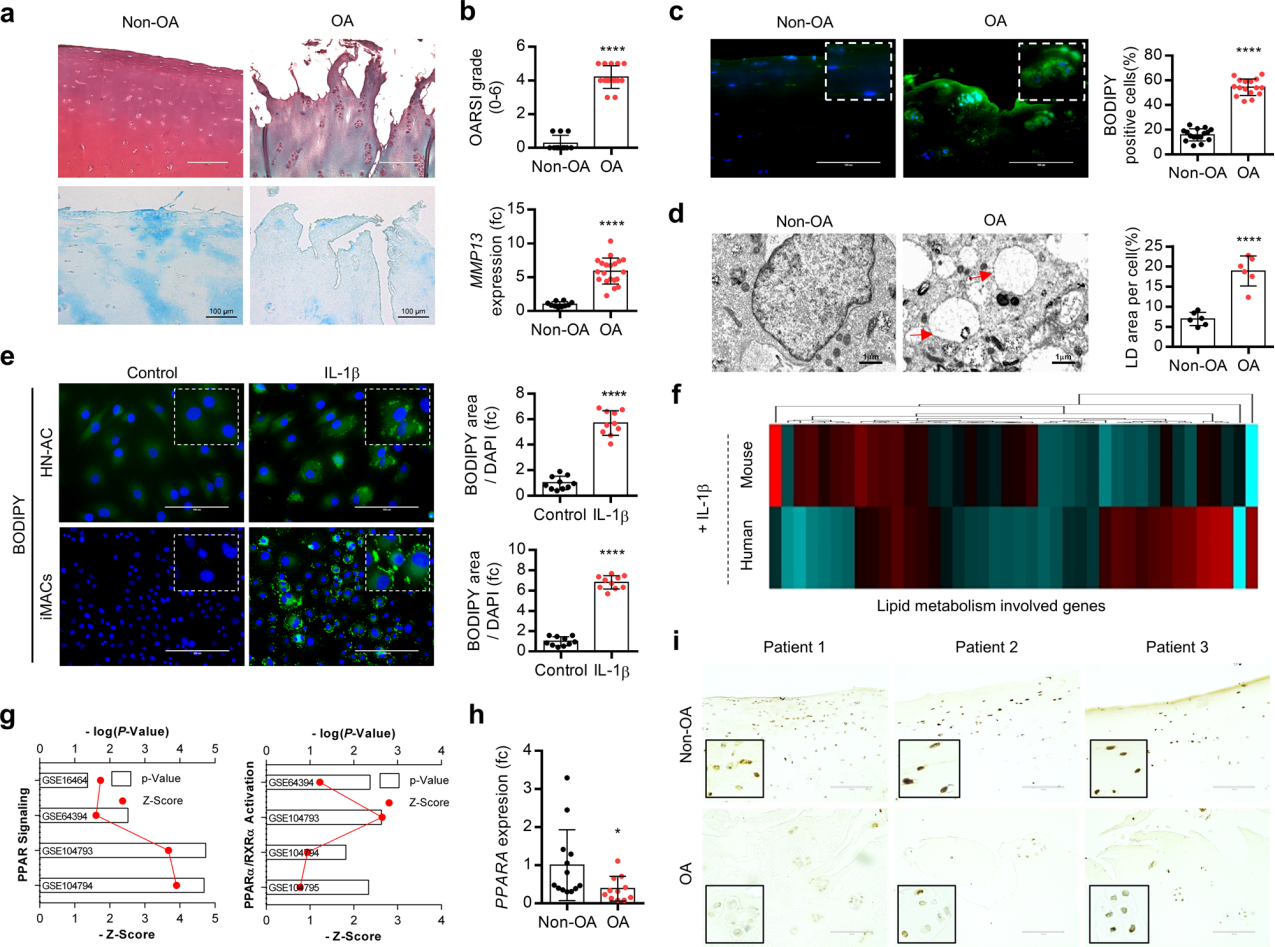
PPARα is responsible for lipid deposition (LD) during OA pathogenesis. **a** Safranin O and Alcian blue staining in non-OA and OA cartilages(non-OA, *n* = 11; OA, *n* = 15). Scale bar, 100 μm. **b** The degree of cartilage degradation is quantified according to OARSI grade. *MMP13* mRNA transcription level in non-OA and OA chondrocytes (non-OA, *n* = 10; OA, *n* = 19), *P* < 0.0001. **c** LD is stained by BODPY (green) in intact cartilage isolated from patients (non-OA, *n* = 16) and damaged region (OA, *n* = 20). Scale bar, 200 μm. *P* < 0.0001. **d** Transmission electron micrographs of non-OA (*n* = 6) and OA (*n* = 6) chondrocytes a*n*d LD area were quantified, *P* < 0.0001. Red arrow indicates lipid droplet. Scale bar, 1 μm. **e** BODIPY staining of immature murine articular chondrocytes (iMACs) and normal human chondrocytes (HN-AC) in the absence or presence of 5 ng/ml IL-1β (*n* = 3), *P* < 0.0001. **f** Expression of genes associated with lipid metabolism in IL-1β-treated HN-AC and WT iMACs. Heatmap analysis was performed using a PermutMatrix-1.9.3. **g** Canonical pathway analysis of GSE16464 (human OA chondrocytes), GSE64394 (human OA chondrocytes), GSE104793 (IL-1β-treated primary mouse articular chondrocytes), GSE104794 (HIF-2α-overexpressed primary mouse articular chondrocytes), GSE104795 (ZIP8-overexpressed primary mouse articular chondrocytes). **h** The expression level of *PPARA* in non-OA and OA chondrocytes (non-OA, *n* = 13, OA, *n* = 11), *P* = 0.0483. **i** Immunohistochemistry of PPARA in non-OA and OA cartilage section of OA patients. The representative region is magnified as dashed line box. Scale bar, 100 μm. Values are means ± SD. Unpaired Student’s *t* test (**b**–**e**, **h**) was used for statistical analysis. *P < 0.05; ****P < 0.0001.

The cartilage of OA patients undergo total knee replacement (TKR) surgery was divided as relatively healthy (non-OA) or severely damaged (OA) region and cartilage degeneration in OA cartilage assessed by Safranin O or Alcian blue staining (Fig. 1a) and Osteoarthritis Research Society International (OARSI) grading^34,35^ (Fig. 1b). We also confirmed the increased expression of matrix metalloproteinase (MMP) 13 in OA cartilage. The LD accumulation in chondrocytes was assessed as staining with BODIPY, a fluorescent probe for neutral lipid or transmission electron microscopy (TEM) analysis (Fig. 1c, d). In this study, to avoid the possible influence or effects of metabolic diseases, sex, and aging in cartilage environment, we studied cartilage tissues in normal BMI (<25) and 50–60-year-old male without hypertension and diabetes mellitus. Consistent with this observation, exposure of interleukin (IL)-1β into human normal articular chondrocyte (HN-AC) cell line and immature murine articular chondrocytes (iMACs) also dramatically induced LD accumulation (Fig. 1e). We also observed a significant increase in the number of BODIPY-positive cells in the cartilage of OA mice induced by destabilization of medial meniscus (DMM) surgery^36^ compared to control cartilage at 8 weeks post surgery (Supplementary Fig. 1). The profile of genes in lipid metabolism using human chondrocytes and iMACs treated with IL-1β (Fig. 1f) as well as GSE dataset (GSE16464; human OA chondrocytes, GSE64394; human OA chondrocytes, GSE104793; IL-1β-treated primary mouse articular chondrocytes, GSE104794; HIF-2α-overexpressed primary mouse articular chondrocytes, GSE104795; ZIP8-overexpressed primary mouse articular chondrocytes) (Fig. 1g) indicated the involvement of peroxisome proliferator-activated receptor (PPAR) signaling in OA pathogenesis. The expression level of PPARα was significantly decreased in chondrocytes and cartilages of OA patients (Fig. 1h, i) and DMM-induced mouse cartilages at post surgery of 4- and 6 weeks (Supplementary Fig. 2).

**Fig. 2:**
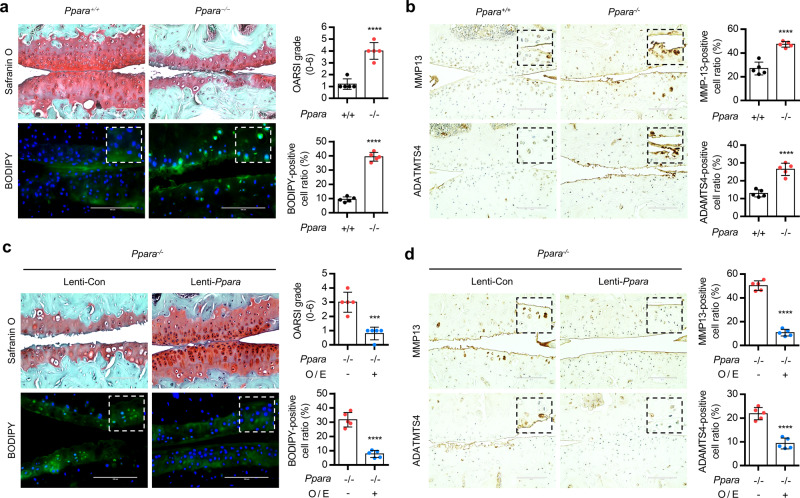
PPARα deficiency is involved in OA pathogenesis. **a** Safranin O or BODIPY staining in the cartilage of *Ppara*^+/+^ and *Ppara*^−/−^ mice at 8 weeks post surgery. The degree of cartilage degradation is quantified according to OARSI grade and BODIPY-positive cells were indicated by bar–dot plot (*n* = 5), *P* < 0.0001. Scale bar, 100 μm. **b** Immunohistochemistry of MMP13 and ADAMTS4 in *Ppara*^+/+^ and *Ppara*^−/−^ mice at 8 weeks post surgery. Scale bar, 100 μm. Positive cells of MMP13 and ADAMTS4 were indicated by bar–dot plot (*n* = 5), *P* < 0.0001. **c** Safranin O and BODIPY staining in the cartilage of DMM-induced *Ppara*^+/+^ and *Ppara*^−/−^ treated with Lenti− *Ppara*. Scale bar, 200 μm. The OARSI grade, the thickness of the tibia, and BODIPY-positive cells are indicated by graph (*n* = 5), *P* = 0.0004 for OARSI grade, *P* < 0.0001 for BODIPY. **d**, Immunostaining of MMP13 and ADAMTS4 in the cartilage of DMM-induced *Ppara*^+/+^ and *Ppara*^−/−^ treated with Lenti− *Ppara*. Positive cells of MMP13 and ADAMTS4 were indicated by bar–dot plot (*n* = 5), *P* < 0.0001. Values are means ± SD. Unpaired Student’s *t* test (**a**–**d**) was used for statistical analysis. ***P < 0.001; ****P < 0.0001.

To confirm the involvement of PPARα in the OA pathogenesis, iMACs were treated with fenofibrate, a potent agonists for PPARα, or GW6471, the specific PPARα inhibitor, or introduced by small interference RNA specific to PPARα (si*Ppara*) in the presence or absence of IL-1β. Exposure of fenofibrate into iMACs significantly reduced the expression level of matrix metalloproteinase (*Mmp*)-*9*, *Mmp13*, a disintegrin and metalloproteinase with thrombospondin motifs (*Adamts*)-*4*, and -*5* and increased cartilage matrix synthesis with a significant reduction of LD accumulation (Supplementary Fig. 3a–c). In addition, LD accumulation in OA chondrocytes was also significantly reduced by the exposure of fenofibrate (Supplementary Fig. 3d). On the other hand, exposure of GW6471 or introduction of si*Ppara* into IL-1β-treated iMACs was stimulated the expression of *Mmp9*, *Mmp13*, *Adatms4*, or *Adamts5*. LD accumulation was also significantly increased by treatment of GW6471 or the introduction of si*Ppara* in IL-1β-treated iMACs. Global deletion of PPARα (*Ppara*^*−/−*^) did not change the skeletal development as assessed by alcian blue/alizarin red staining on postnatal day 0 (P0) and the thickness of tibial plateau compared to the cartilage of *Ppara*^*+/+*^ mice (Supplementary Fig. 4a, b). The presence of sulfated proteoglycans in the culture of *Ppara*^*−/−*^ iMACs was not different from the culture of *Ppara*^*+/+*^ iMACs (Supplementary Fig. 4c). However, a significant increase in LD accumulation and cartilage degradation and (Fig. 2a) and increased levels of MMP13 and ADAMTS4 (Fig. 2b) were observed in DMM-induced *Ppara*^*−/−*^ cartilage. Restoration of *Ppara* using lentiviruses containing *Ppara* (Lenti- *Ppara*) into joint cavity of *Ppara*^*−/−*^ mouse reduced the cartilage degradation, decreased number of BODIPY-positive cells, and decreased expression level of MMP13 and ADAMTS4 in *Ppara*^*−/−*^ cartilage (Fig. 2c, d).

### Decreased level of ACOT12 is responsible for the cartilage degradation by *Ppara* deficiency

**Fig. 3:**
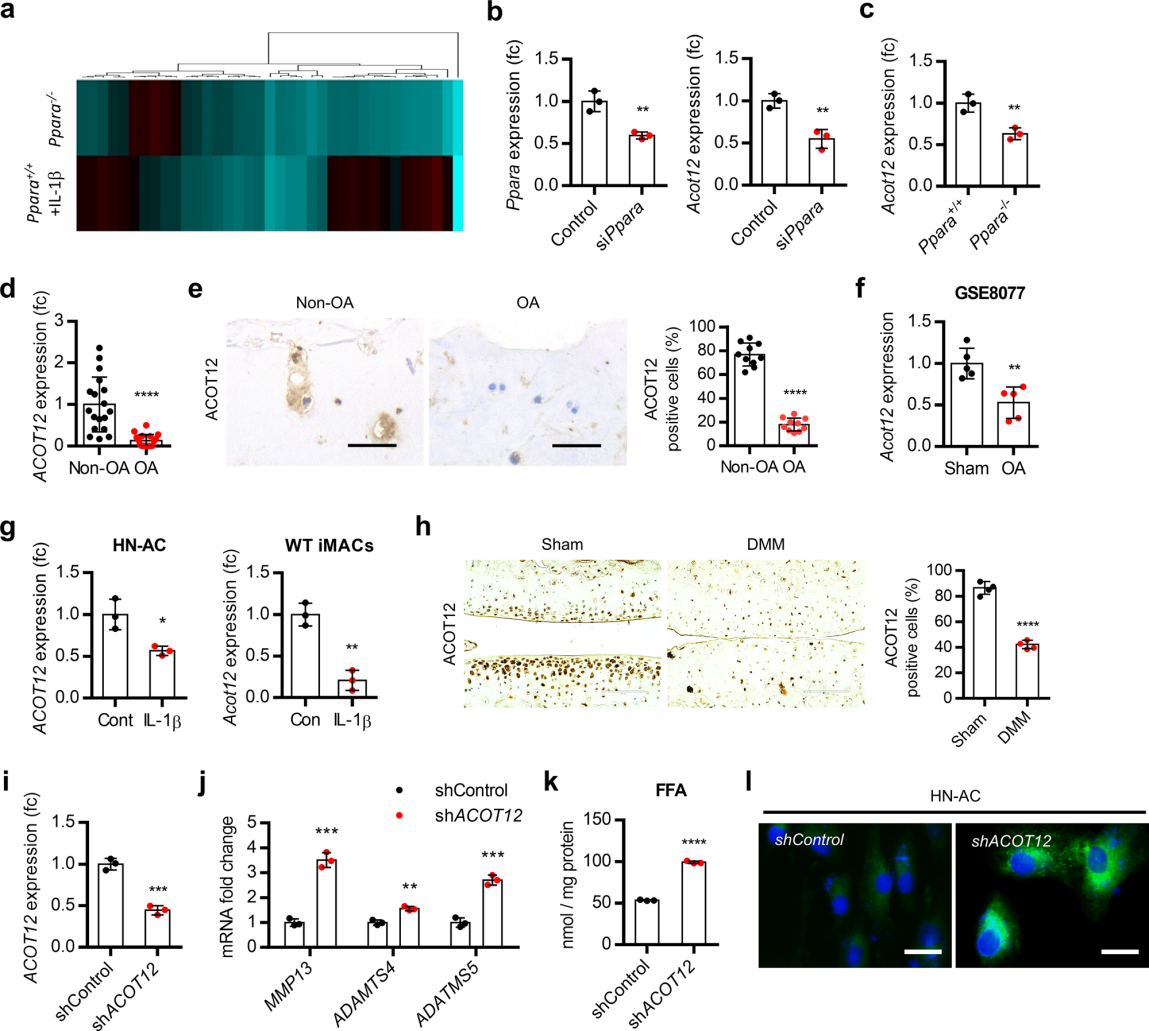
ACOT12 is a major regulatory factor in OA pathogenesis induced by PPARα deficiency. **a** Heatmap analysis of lipid metabolism genes in *Ppara*^*+/+*^ and *Ppara*^*−/−*^ iMACs in the presence of IL-1β. Heatmap analysis was performed using a PermutMatrix-1.9.3. **b** Expression level of *Acot12* in iMACs introduce siRNA specific to *Ppara* (si*Ppara*). Knockdown efficiency of si*Ppara* was confirmed by qRT-PCR (*n* = 3), *P* = 0.0056 for *Ppara*, *P* = 0.0050 for *Acot12*. **c** Expression level of Acot12 in *Ppara*^*+/+*^ and *Ppara*^−*/−*^ iMACs (*n* = 3), *P* = 0.0077). **d** Expression level of *ACOT12* in non-OA (*n* = 18) and OA (*n* = 18) chondrocytes (*P* < 0.0001). **e** Immunohistochemistry of ACOT12 in non-OA and OA cartilage (*P* < 0.0001). Scale bars, 50 μm. **f** Expression of Acot12 in GSE8077 of rat OA articular chondrocytes (*n* = 5), *P* = 0.0041. **g** Expression level of ACOT12 in HN-AC (*n* = 3) and WT iMACs (*n* = 3), *P* = 0.0170 for HN-AC, *P* = 0.0017 for WT iMACs. **h** Immunohistochemistry of ACOT12 in OA-induced mouse cartilage (*n* = 4), *P* < 0.0001. Scale bars, 100 μm. **i** shACOT12 was introduced into HN-AC (*n* = 3). Knockdown efficiency of shAOCT12 was confirmed by qRT-PCR (*n* = 3), *P* = 0.0004. **j** Expression of *Mmp13*, *Adamts4* and *−5* in shACOT12-introduced HN-AC (*n* = 3), *P* = 0.0002 for *Mmp13, P* = 0.0018 for *Adamts4*; *P* = 0.0004 for *Adamts5*. **k** Cellular level of FFA in sh*ACOT12*-introduced HN-AC (*n* = 3), *P* < 0.0001. **l** BODIPY staining in shACOT12-introduced HN-AC cells. Scale bars, 20 μm. Values are means ± SD. Unpaired Student’s *t* test (**b**–**i**, **k**) or multiple *t* test followed by Holm–Sidak method (**j**) were used for statistical analysis. *P < 0.05; **P < 0.01; ***P < 0.001; ****P < 0.0001.

To elucidate the responsible factors in cartilage degradation induced by PPARα deficiency, we analyzed the alteration of expression levels in downstream target genes of PPARα using *Ppara*^*−/−*^ iMACs and IL-1β-treated *Ppara*^*+/+*^ iMACs and identified ACOT12 as a key regulator factor in this (Fig. 3a). The expression level of *Acot12* was significantly increased by the introduction of si*Ppara* into iMACs of *Ppara*^*+/+*^ mice as well as in iMACs of *Ppara*^*−/−*^ mice (Fig. 3b, c). Moreover, decreased *ACOT12* level and decreased number of ACOT12-positive cells were observed in OA chondrocytes and OA cartilage (Fig. 3d, e). The protein level of ACOT12 in articular chondrocyte was confirmed by western blotting using chondrocyte progenitor cell (CPC) and iMACs (Supplementary Fig. 5a). Consistent with our data, analysis of GSE8077 of rat articular OA chondrocytes also showed the decreased level of ACOT12 in OA chondrocytes (Fig. 3f). Exposure of IL-1β into human normal chondrocytes (HN-AC) or iMACs significantly reduced the expression level of ACOT12 (Fig. 3g). Decreased ACOT12-positive cells were observed in the cartilage of DMM-induced mice (Fig. 3h and Supplementary Fig 5b). Introduction of short hairpin RNA against ACOT12 (sh*Acot12*) into HN-AC accumulated free fatty acid and LD with significant increases of *MMP13*, *ADAMTS4*, and *ADAMTS 5* level (Fig. 3j–l). These data suggest that ACOT12 may act as a key regulator in the cartilage degradation induced by PPARα deficiency.

### *Acot12*^*−/−*^ mice display the typical OA characteristics by activating de novo lipogenesis

To investigate the functional role of ACOT12 in vivo, we generated *Acot12* knockout (KO) mice by germline transmission of RNA-guided endonuclease (RGEN)-induced mutant *Acot12* alleles (Supplementary Fig. 6a). Global deletion of *Acot12* did not alter bone development such as endochondral ossification and postnatal limb length (Supplementary Fig. 6b). Unlike WT (*Acot12*^*+/+*^) mice, homozygous KO (*Acot12*^*−/−*^) mice showed signs of spontaneous cartilage destruction at 12 months of age, not at 3 months of age compared to wild-type (*Acot12*^*+/+*^) mice (Supplementary Fig. 6c).

**Fig. 4:**
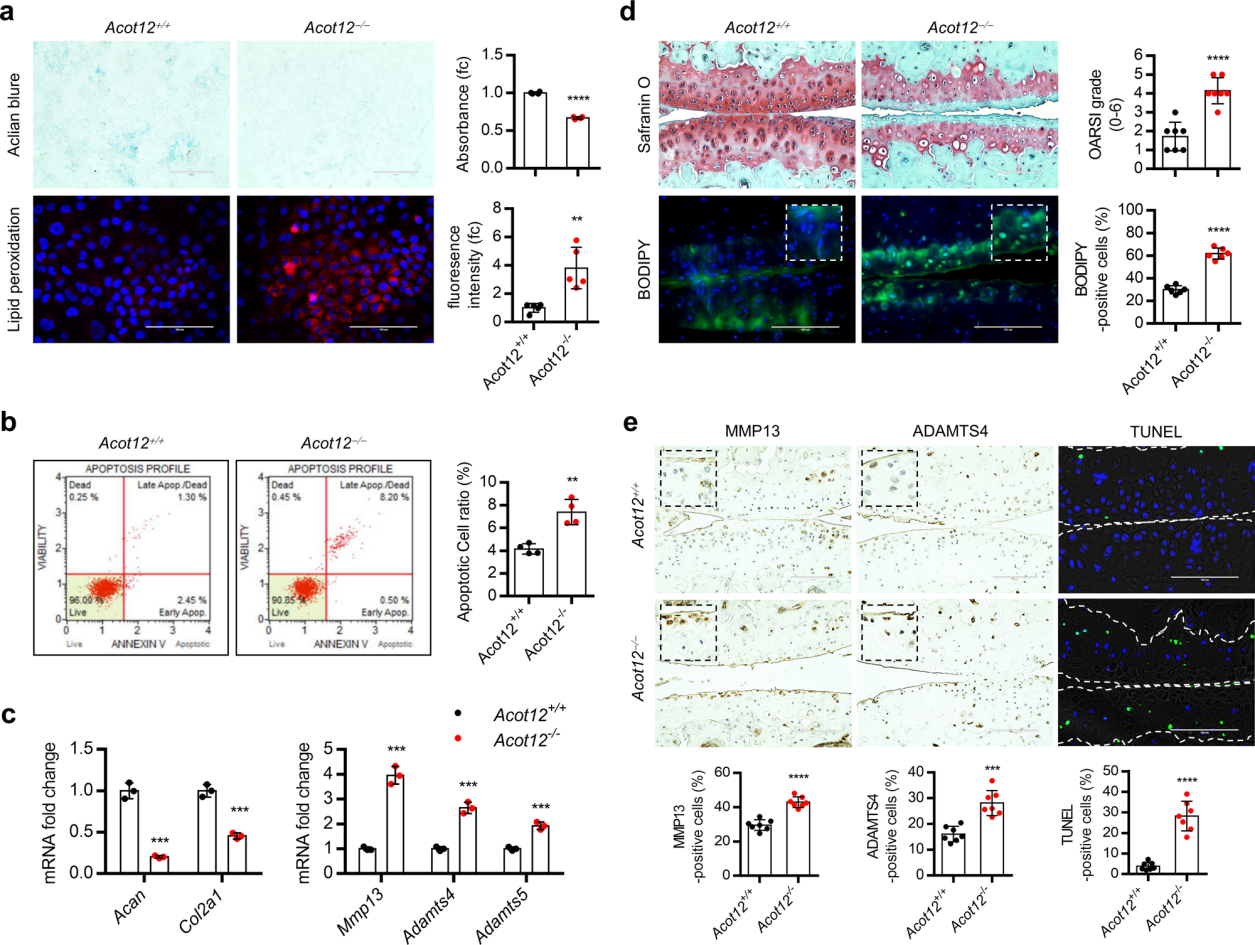
ACOT12 is involved in maintaining cartilage homeostasis. **a** Alcian blue (*n* = 3) and lipid peroxidation (*n* = 5) staining in *Acot12*^*+/+*^ and *Acot12*^*−/−*^ iMACs treated with 5 ng/ml IL-1β, *P* < 0.0001 for Alcian blue, *P* = 0.0030 for lipid peroxidation. **b** Analysis of apoptotic cell death (*n* = 4) in *Acot12*^*+/+*^ or *Acot12*^*−/−*^ iMACs, *P* = 0.0016. **c** Expression level of *Acan* (*P* = 0.0001), *Col2a1* (*P* = 0.0004), *Mmp13* (*P* = 0.0002), *Adamts4* (*P* = 0.0003), and *Adatms5* (*P* = 0.0008) in *Acot12*^*+/+*^ and *Acot12*^*−/−*^ iMACs treated with 5 ng/ml IL-1β (*n* = 3). **d** Safranin O and BODIPY staining in cartilage of DMM-induced *Acot12*^*+/+*^ and *Acot12*^*−/−*^ mice at 8 weeks. The degree of cartilage degradation is quantified according to OARSI grade (*n* = 7), *P* < 0.0001. Scale bar, 100 μm. BODIPY-positive cells are indicated by bar–dot plot (*n* = 6), *P* < 0.0001. **e** Immunohistochemistry of MMP13 and ADAMTS4 and TUNEL staining in the cartilage section of *Acot12*^*+/+*^ and *Acot12*^*−/−*^ mice (*n* = 7). MMP13 (*P* < 0.0001), ADAMTS4 (*P* = 0.0001), and TUNEL-positive cells (*P* < 0.0001) were indicated by bar–dot plot. Unpaired Student’s *t* test (**a**, **b**, **d**, **e**) or multiple *t* test followed by Holm–Sidak method (**c**) were used for statistical analysis. **P < 0.01, ***P < 0.001, ****P < 0.0001.

Decreased level of cartilage matrix, increase level of lipid peroxidation (Fig. 4a), stimulation of apoptotic cell death (Fig. 4b), decreased level of *Acan* and *Col2a1*, increased level of *Mmp13*, *Adamts4* and −*5* (Fig. 4c) were observed in *Acot12*^*−/−*^ iMACs compared to *Acot12*^*+/+*^ iMACs. Severe cartilage destruction and increased number of BODIPY-positive cells were observed in DMM-induced *Acot12*^*−/−*^ mice at 8 weeks of post surgery compared to sham-operated mice (Fig. 4d). MMP13- and ADAMTS4- and TUNEL-positive cells were also significantly increased in DMM-induced *Acot12*^*−/−*^ cartilage (Fig. 4e).

**Fig. 5:**
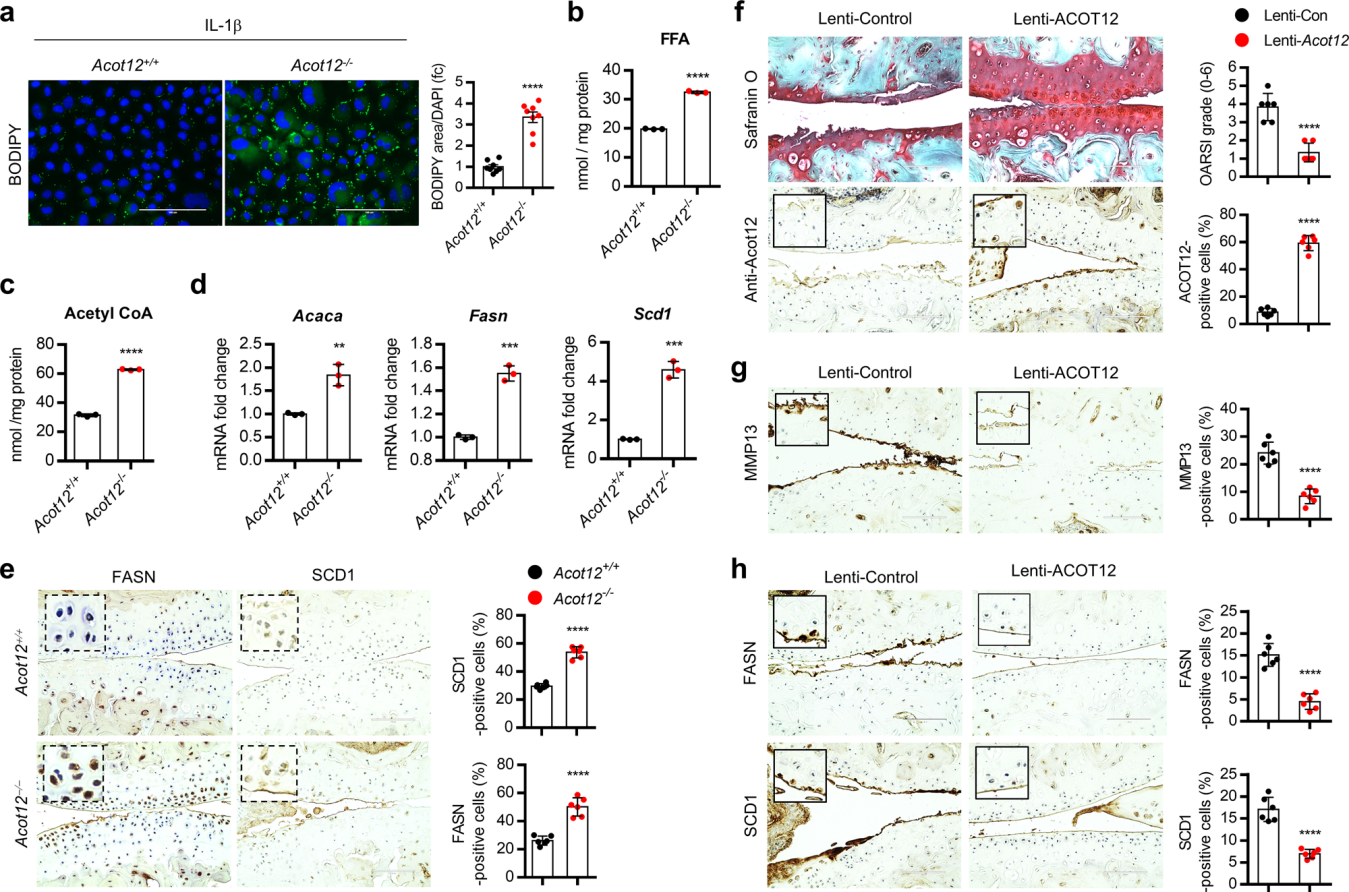
*Acot12*-downregulation is involved in OA pathogenesis via DNL. **a** BODIPY^493/503^ staining and positive area in *Acot12*^*+/+*^ or *Acot12*^−*/*−^ iMACs in the presence of IL-1β (*n* = 8) *P* < 0.0001. Scale bar, 100 μm. **b** Cellular level of FFA in *Acot12*^*+/+*^ or *Acot12*^*−/−*^ iMACs (*n* = 3), *P* < 0.0001). **c** Cellular level of a**c**etyl-CoA in *Acot12*^*+/+*^ or *Acot12*^*−/−*^ iMACs (*n* = 3), *P* < 0.0001. **d** Expression level of *Acaca* (*P* = 0.0032), *Fasn* (*P* = 0.0002), and *Scd1* (*P* = 0.0001) in *Acot12*^*+/+*^ or *Acot12*^*−/−*^ iMACs (*n* = 3). **e** Immunohistochemistry a*n*d positive cell counting of FASN and SCD1 in *Acot12*^*+/+*^ or *Acot12*^*−/−*^ mice (*n* = 6), *P* < 0.0001. Scale bar, 100 μm. **f** Safranin O and ACOT12 staining in cartilage of DMM-induced OA mouse with lentiviral *ACOT12* (*n* = 6). The degree of cartilage degradation quantified according to OARSI grade and *ACOT12*-positive cell are indicated by bar–dot plot (*P* < 0.0001). Scale bar, 100 μm. **g** Immunohistochemistry of MMP13 in cartilage of DMM-induced mouse with lentiviral *ACOT12* (*n* = 6), *P* < 0.0001. **h** Immunohistochemistry of FASN and SCD1 in cartilage of DMM-induced mouse with lentiviral *ACOT12* (*n* = 6), *P* < 0.0001. Values are means ± SD. An unpaired Student’s *t* test (**a**–**h**) was used for statistical analysis. **P < 0.01, ***P < 0.001, ****P < 0.0001.

Increased number of BODIPY-positive cells (Fig. 5a) and increased level of free fatty acid (Fig. 5b) were observed in IL-1β-treated *Acot12*^*−/−*^ iMACs compared to IL-1β-treated *Acot12*^*+/+*^ iMACs. Increased level of acetyl-CoA was observed in *Acot12*^*−/−*^ iMACs (Fig. 5c). Moreover, increased level of genes in de novo lipogenesis (DNL) such as acetyl-Coenzyme A carboxylase alpha (*Acaca*), fatty acid synthase (*Fasn*), stearoyl-CoA desaturase (*Scd*)1 was also observed in *Acot12*^*−/−*^ iMACs (Fig. 5d). Increased number of FASN- and SCD1-positive cells were observed in *Acot12*^*−/−*^ cartilage (Fig. 5e) as well as OA chondrocyte and cartilage of patients (Supplementary Fig. 7).

To determine whether the recovery of *ACOT12* into chondrocyte could prevent cartilage degradation, *ACOT12*-encoding pcDNA (pcDNA-*Acot12*) were introduced into *Acot12*^*+/+*^ iMACs or *Acot12*^*−/−*^ iMACs in the presence of IL-1β or *ACOT12*-encoding lentivirus (Lenti-HA-*Acot12*) were injected into knee joint cavity of *Acot12*^*−/−*^ mouse. With the restoration of ACOT12, a significant reduction in triglyceride (TG), decreased number of BODIPY-positive cells, and increased cartilage matrix were observed both in IL-1β-treated *Acot12*^*+/+*^ iMACs or *Acot12*^*−/−*^ iMACs (Supplementary Figs. 8 and 9). Intra-articular injection of Lenti-HA-*Acot12* into *Acot12*^*−/−*^ mouse joint resulted in a decrease in cartilage degradation with an increase in the number of *Acot12*-positive cells (Fig. 5f). In addition, the expression level of MMP13, FASN, and SCD1 was also inhibited by intra-articular injection of Lenti-HA-*Acot12* in DMM-induced mice (Fig. 5h).

### Accumulation of acetyl-CoA by ACOT12 deficiency stimulates DNL during OA pathogenesis

Because articular cartilage does not have blood vessels, nerves, or lymphatics unlike most tissue, chondrocytes mainly depend on anaerobic metabolism using glucose as an important fuel to maintain cartilage homeostasis. Several reports suggested that the glycolytic metabolic pathway converting glucose into lactate when limited amounts of oxygen are available, or pyruvate which then enters the Krebs cycle is upregulated during OA pathogenesis^7,37,38^. In silico analysis of GSE57218, GSE104794 and GSE104795 indicated a significant increase in glycolysis during OA pathogenesis (Supplementary Fig. 10a). Enrichment plot of KEGG and GDEA analysis from RNA-seq data of OA patient (OA vs non-OA) also suggested glycolysis as one of the enriched signaling pathways in OA pathogenesis (Supplementary Fig. 10b). Consistent with these data, *Acot12*^*−/−*^ iMACs showed activated glycolysis pathway and increased level of glycolysis genes such as *hexokinase 2* (*HK2*), *phosphofructokinase* (*PFKP*), *phosphoglycerate mutase 2* (*PGAM2*) and *phosphoenolpyruvate carboxykinase* (*PCK*) (Supplementary Fig. 10c, d). Inhibition of glycolysis by 2-deoxy-d-glucose (2DG) suppressed LD accumulation in the presence of IL-1β without significant differences in chondrocyte apoptosis (Supplementary Fig. 11). These data suggested that LD accumulated in OA chondrocytes may derive from glycolysis, not from free FA in the extracellular environment.

**Fig. 6:**
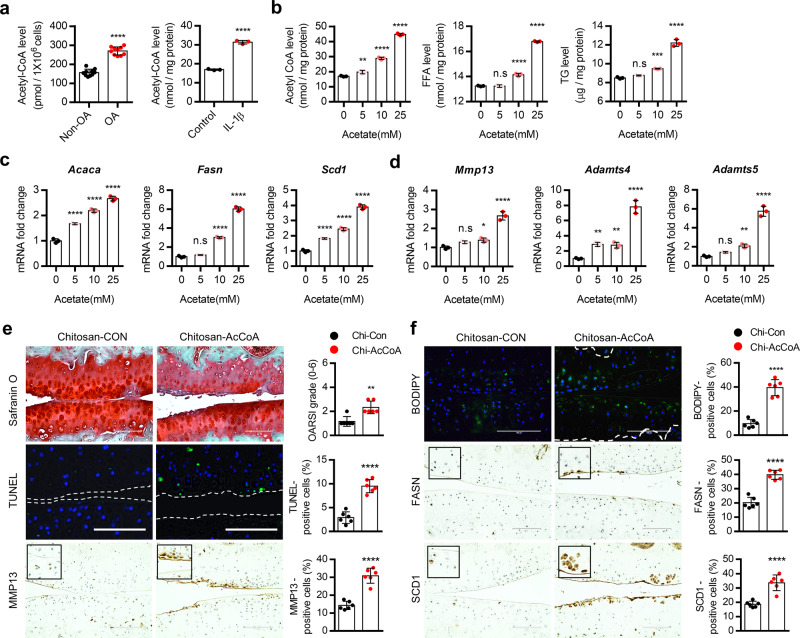
Acetyl-CoA overload is responsible for stimulation of de novo lipogenesis during OA pathogenesis. **a** Cellular level of acetyl-CoA in non-OA (*n* = 10) and OA (*n* = 10) chondrocytes (left panel), *P* < 0.0001, and iMACs-treated w/wo IL-1β (right panel), *P* < 0.0001. **b** Cellular level of acetyl-CoA (0 vs. 5 mM acetate, *P* = 0.0072, 10 mM acetate, *P* < 0.0001, 25 mM acetate, *P* < 0.0001) FFA (0 vs. 5 mM acetate, *P* = 0.9928, 10 mM acetate, *P* < 0.0001, 25 mM acetate, *P* < 0.0001), and TG (0 vs. 5 mM acetate, *P* = 0.2767, 10 mM acetate, *P* = 0.0006, 25 mM acetate, *P* < 0.0001) with sodium acetate treatment (*n* = 3). **c** Expression level of *Acaca* (*P* < 0.0001), *Fasn* (0 vs. 5 mM acetate, *P* = 0.3177, 10 mM acetate, *P* < 0.0001, 25 mM acetate, *P* < 0.0001 for 25 mM) and *Scd1* (*P* < 0.0001) with sodium acetate treatment (*n* = 3). **d** Expression level of *Mmp13* (0 vs. 5 mM acetate, *P* = 0.0819, 10 mM acetate, *P* = 0.0199, 25 mM acetate, *P* < 0.0001), *Adamts4* (0 vs. 5 mM acetate, *P* = 0.0038, 10 mM acetate*, P* = 0.0053, 25 mM acetate, *P* < 0.0001), and *Adamts5* (0 vs. 5 mM acetate, *P* = 0.2391, 10 mM acetate, *P* = 0.0045, 25 mM acetate, *P* < 0.0001) with sodium acetate treatment. **e** Safranin O staining, TUNEL analysis, and immunohistochemistry of MMP13 in mouse cartilage injected with chitosan (*n* = 6) or chitosan-AcCoA (*n* = 6). The degree of cartilage degradation quantified according to OARSI grade (*P* = 0.0016) and TUNEL- (*P* < 0.0001) and MMP13-positive cell (*P* < 0.0001) are indicated by bar–dot plot. Scale bars, 100 μm. **f** BODIPY staining and immunohistochemistry of FASN and SCD1 in mouse cartilage injected with chitosan or chitosan-AcCoA. Scale bars, 100 μm. BODIPY- (*P* < 0.0001), FASN- (*P* < 0.0001), or SCD1-positive cells (*P* < 0.0001) indicated by bar–dot plot (*n* = 6). Values are means ± SD. Unpaired Student’s *t* test (**a**, **e**, **f**) or one-way ANOVA followed by Dunnett’s multiple comparisons test (**b**–**d**) were used for statistical analysis. n.s, not significant *P* >= 0.05; *P < 0.05; **P < 0.01; ***P < 0.001; ****P < 0.0001.

In addition, analysis from RNA-seq data of OA patient (OA vs non-OA) showed that expression levels of genes participating in lipogenic pathways such as FA synthesis (ACLY, ACSS2, and ACCL), FA elongation (SCD1, ELOVL5, and ELOVL6), and FA desaturation (FADS2 and FADS3) were significantly increased in OA patient chondrocytes (Supplementary Fig. 12) suggesting that LD in OA chondrocytes is possibly due to activation of de novo lipogenic pathway (DNL). Since DNL initiated with the conversion of acetyl-CoA into malonyl-CoA, these data suggested that increased acetyl-CoA level by ACOT12 deficiency could activate DNL. Among three major enzymes, ACLY, ACSS2, or ACOT12, involved in the regulation of acetyl-CoA pool^33,39,40^ (Supplementary Fig. 13a), expression levels of *ACLY* and *ACSS2* which are involved in the production of cytosolic acetyl-CoA were significantly increased whereas expression level of *ACOT12* which is involved in the hydrolysis of cytosolic acetyl CoA was significantly decreased by IL-1β-treated HC-N cells (Supplementary Fig. 13b) and DMM-induced cartilage (Supplementary Fig. 13c) indicating the accumulation of acetyl-CoA during OA pathogenesis. To investigate the effect of acetyl-CoA accumulation on cartilage homeostasis, iMACs were treated with acetate in a dose-dependent manner. With increasing the concentration of acetate, increased cellular level of acetyl-CoA, FFA, and TG (Fig. 6b), increased expression level of genes in DNL such as *Acaca*, *Fasn*, and *Scd1* (Fig. 6c) and in cartilage-degrading enzyme such as *Mmp13*, *Adamts4*, and *−5* (Fig. 6d) were observed. Furthermore, acetyl-CoA-conjugated chitosan complex (Supplementary Fig. 14a, b) was delivered into articular chondrocyte twice per week by intra-articular injection after DMM surgery^41,42^ and delivery efficiency was confirmed by fluorescein isothiocyanate (FITC)-labeled chitosan (Supplementary Fig. 14c). In the cartilage injected with acetyl CoA-conjugated chitosan complex (Chitosan-AcCoA), cartilage degradation as assessed by safranin O staining and the number of BODIPY- and TUNEL-positive cells were significantly increased compared to cartilages injected chitosan alone (Chitosan-Con) and sham-operated cartilage (Supplementary Fig. 15). Increased cartilage degradation and increased number of TUNEL- and MMP13-positive cells (Supplementary Fig. 15a, b) as well as the increased number of BODIPY-, FASN-, and SCD1-positive cells were observed with the delivery of Chitosan-AcCoA into *Acot12*^*+/+*^ mice twice per week by intra-articular injection (Fig. 6e, f).

### ACOT12 overexpression prevents OA pathogenesis induced by PPARα deficiency

**Fig. 7:**
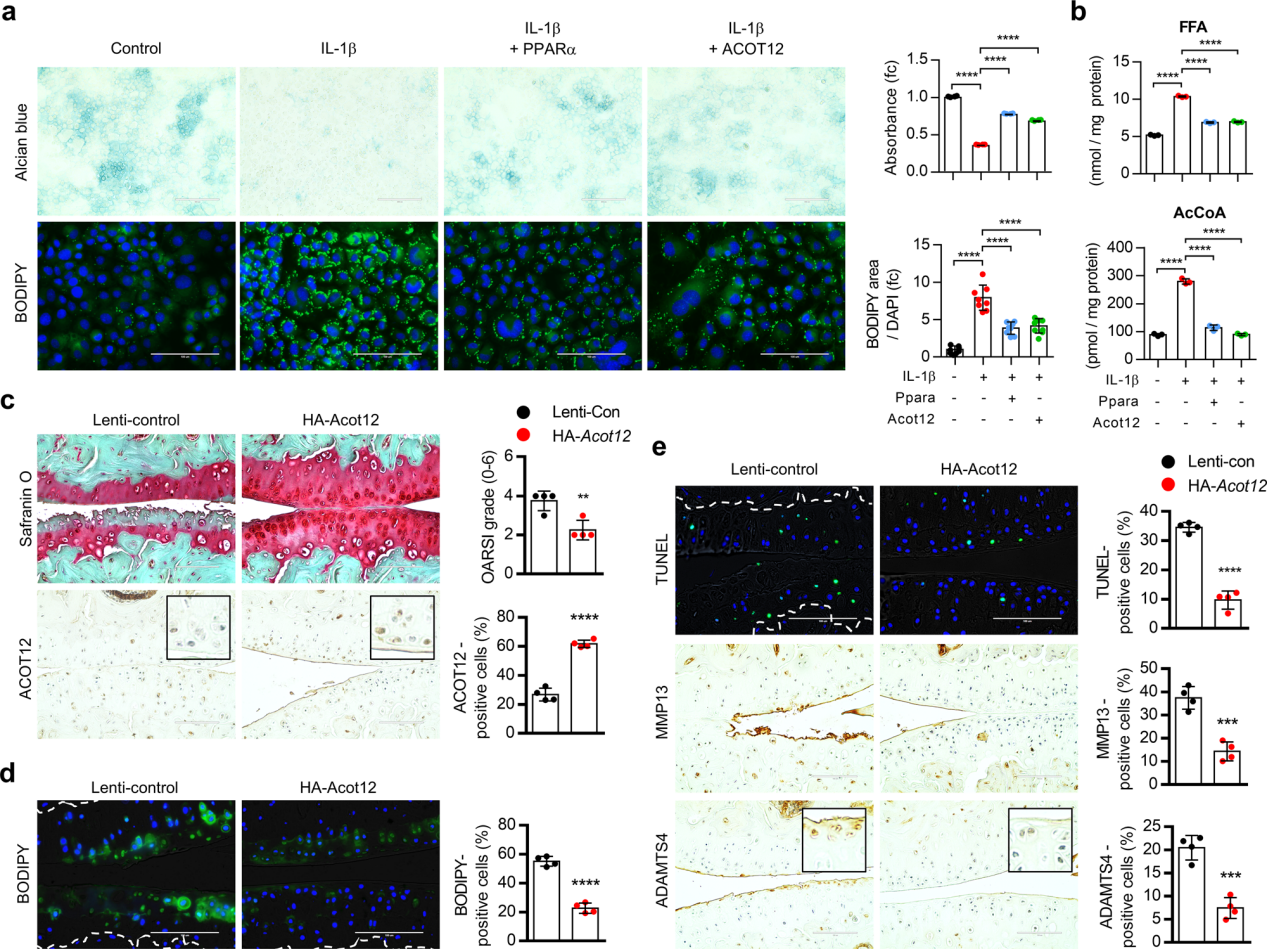
ACOT12 overexpression alleviates lipid accumulation and OA pathogenesis induced by PPARα deficiency. **a** Alcian blue (*n* = 3) and BODIPY (*n* = 8) staining of *Ppara*^*−/−*^ iMACs introduced by PPARα or ACOT12 expression vector in the absence or presence of IL-1β, *P* < 0.0001 for Alcian blue, *P* < 0.0001 for BODIPY. Scale bars, 100 μm. **b** Cellular level of FFA (*P* < 0.0001) and acetyl-CoA (*P* < 0.0001) in *Ppara*^*−/−*^ iMACs introduced by PPARα or ACOT12 expression vector in the absence or presence of IL-1β. **c** Safranin O staining and ACOT12 staining in the **c**artilage of *Ppara*^−*/−*^ mice transfected with lentivirus containing HA-tagged Acot12 (HA-Acot12). Scale bars, 100 μm. OARSI grade (*P* = 0.0054) and ACOT12-positive cells (*P* < 0.0001) indicated by bar–dot plot (*n* = 4). **d** BODIPY^483/503^ staining of in cartilage of *Ppara*^−*/−*^ mice transfected with HA-ACOT12 (*n* = 4), *P* < 0.0001. Subchondral bone indicated with white line. Scale bars, 100 μm. **e** TUNEL (*P* < 0.0001)-, MMP13 (*P* = 0.0004)-, ADAMTS4 (*P* = 0.0003) staining in cartilage of *Ppara*^*−/−*^ mice transfected with HA-ACOT12 (*n* = 4). Subchondral bone indicated with white line in TUNEL staining. Scale bars, 100 μm. Values are means ± SD. Unpaired Student’s *t* test or one-way ANOVA (**c**–**e**) followed by Tukey’s multiple comparisons test (**a**, **b**) were used for statistical analysis. **P < 0.01; ***P < 0.001; ****P < 0.0001.

To confirm the involvement of ACOT12 in the OA pathogenesis induced by PPARα deficiency, lentivirus containing HA-tagged ACOT12 (HA-*Acot12*) were transfected into *Ppara*^−*/−*^ iMACs or injected into the cartilage of *Ppara*^*−/−*^ mouse. In *Ppara*^−*/*−^ iMACs, cartilage matrix was increased and LD accumulation was decreased by introduction of ACOT12 same as shown in the restoration of PPARα (Fig. 7a). Increased level of FFA and acetyl-CoA by IL-1β was also significantly decreased with introduction of ACOT12 as well as the restoration of PPARα (Fig. 7b). Moreover, decreased levels of MMP13, ADAMTS-4, and −5 were observed with introduction of ACOT12 into *Ppara*^*−/−*^ iMACs (Supplementary Fig. 16a). Consistent with this, injection of HA-Acot12 into the cartilage of *Ppara*^*−/−*^ mice significantly reduced the degradation of cartilage (Fig. 7c and Supplementary Fig. 16b). Increased lipid accumulation and the number of TUNEL-, MMP13-, ADAMTS4-positive cells in *Ppara*^*−/−*^ catilage were significantly decreased by introduction of HA-*Acot12* (Fig. 7d, e). These data suggest that ACOT12 is a major regulatory factor in the OA pathogenesis induced by PPARα deficiency and introduction of exogenous ACOT12 could effectively alleviate cartilage degradation induced by PPARα deficiency.

## Discussion

Fine regulation of metabolism is important for maintaining cartilage homeostasis and impaired metabolism could trigger various pathophysiological situations through stimulation of pro-inflammatory cytokines and biomechanical stress^7,43^. Thus, metabolic reprogramming in chondrocyte from pathological metabolic state to normal metabolic state by certain enzymes could be one of the possible alternative approaches to OA therapeutic intervention. Several metabolic pathways including glycolysis and fatty acid oxidation are associated with OA pathogenesis^7^. Peroxisome proliferator-activated receptor (PPAR)s, members of the nuclear hormone receptor superfamily in mediating the physiological actions of FA and FA derivatives are known to be involved in the OA pathogenesis^44^. Among three PPAR isoforms, PPARα, PPARβ/δ, and PPARγ, PPARγ is the most studied member in OA pathogenesis. PPARγ1, predominantly expressed in human cartilage, is known to have a protective role against OA^45^. Agonists of PPARγ downregulate inflammatory response and prevent cartilage degradation in OA animal models. Recently several studies have reported that PPARα also plays an important role in cartilage homeostasis^46,47^. PPARα activation by its agonist, WY14643, decreases inflammatory and cartilage destructive responses in OA cartilage^47^ suggesting its protective effect on articular cartilage against OA pathogenesis. Here, in this study, we found the protective role of PPARα against cartilage degradation via regulating acetyl-CoA pool through ACOT12 modulation. we also observed that fenofibrate, the agonist for PPARα, reduced the typical characteristics of OA such as increased levels of cartilage-degrading enzymes such as MMP-9, −13, ADMATS-4, and −5 and LD accumulation. Furthermore, we also observed the increase in cartilage degradation and LD accumulation in DMM-induced *Ppara*^*−/−*^ cartilage.

Accumulating evidences are emerging a key role for metabolites such as ATP, acetyl-CoA, and pyruvate in the development and progression of OA^48–50^. Among them, acetyl-CoA is known as a central intermediate and a major substrate in many biological responses^39,51^. The level of acetyl-CoA could be competitively regulated by anabolic and catabolic enzymes such as *ACC1* and *ACAT1* as well as *ACOT12*^30,52,53^, which is indispensable for the control of lipid synthesis^33^. *ACLY* and *ACSS2* are the metabolic enzymes responsible for the synthesis of acetyl-CoA from citrate and acetate, respectively^40,54,55^. ACOT12 is the major enzyme known to hydrolyze cytosolic acetyl-CoA and is expressed mainly in hepatocytes and sparsely in the kidney and intestine^33,56,57^. Among these three enzymes, *ACLY* has been reported as an important regulator for acetyl-CoA accumulation in OA chondrocytes^58^. In this study, we identified *ACOT12* as one of the crucial factors in maintaining cartilage homeostasis by regulating a novel metabolic circuit via acetyl-CoA. We observed a suppressed level of ACOT12 in human OA cartilages and of OA-induced animal cartilages. Germline deletion of *Acot12* spontaneously developed articular cartilage lesions without affecting bone development. Cartilage degradation was exacerbated due to a decreased chondrocyte cellularity by activating *Mmp-13* and increasing lipid depositions (LD) in chondrocytes, particularly through acetyl-CoA accumulation in DMM-induced *Acot12*^*−/−*^ mice.

Then, where do lipid droplets in *Acot12*^*−/−*^ cartilage come from Intracellular lipids can accumulate from an increased lipid influx in blood or stimulation of autophagy or can be synthesized de novo^59^. In this study, we found that de novo lipogenesis (DNL) might be the one key regulatory process for LD accumulation induced by *Acot12* deficiency. In *Acot12*^*−/−*^ cartilage, an increased level of genes in DNL contributing lipid accumulation in chondrocytes was observed and this could lead lipotoxicity and metabolic stress to chondrocytes. Acetyl-CoA is a crucial component in lipid metabolism, as it is a requisite carbon donor in the de novo synthesis of fatty acids and used by acetyl-CoA carboxylase (ACC) to catalyze the synthesis of malonyl-CoA. In *Acot12*^*−/−*^ cartilage, increased level of acetyl-CoA was observed and increased cellular level of acetyl-CoA by treating acetate or delivery of Chitosan-AcCoA into cartilage increased DNL in cartilage and stimulated cartilage degradation by unbalancing between catabolic and anabolic reaction. The recent study also suggests the importance of acetyl-CoA in the OA pathogenesis. Reduced level of acetyl-CoA in cartilage by ACLY deficiency showed a chondroprotective action and resulted less cartilage damage^58^.

Taken together, our study suggested ACOT12 is the main key regulatory factor in PPARα-mediated cartilage homeostasis through regulating acetyl-CoA pool. Increased level of acetyl-CoA by ACOT12 deficiency stimulates DNL pathway and resulted in the stimulation of cartilage degradation. Therefore, targeting ACOT12 could be one of the potent therapeutic approaches for controlling cartilage degradation.

## Methods

### Human OA specimens and primary chondrocyte culture

Human articular cartilage specimens were obtained from patients undergoing total knee replacement (TKR) surgery. Specimens from osteoarthritic cartilages were classified as relatively heathy (non-OA) or severe damaged (OA) region. Human articular cartilage specimens were cut into pieces, and some pieces were immediately fixed by 10% neutral buffered formalin and others used for primary chondrocyte culture. For primary human chondrocyte culture, pieces of each cartilage (OA or non-OA) were digested using 0.06% collagenase (Sigma) and cultured in high glucose DMEM (Gibco) supplemented with 10% fetal bovine serum (FBS), 1× antibiotic–antimycotic (Gibco). The cells were cultured at 37 °C in a humidified atmosphere of 5% CO_2_.

### Animals

Wild-type C57BL/6N mice were purchased from Samtako BioKorea Inc. (Osan, Korea). Eight-week-old male C57BL/6N mice used in DMM surgery and 5-days-old pups used in iMACs culture. All mice were housed at 23 ± 1 °C with 12-h light/dark cycles and a relative humidity of 50 ± 5% with food and water available ad libitum.

### Experimental OA and histology of OA cartilage

Experimental OA was induced by destabilization of the medial meniscus (DMM) surgery using 8-weeks-old male mice. Knee joints were analyzed 8 weeks after DMM surgery. Cartilages were processed for histological and biochemical analyses. Briefly, knee joints were fixed in 4% paraformaldehyde, decalcified in 14% EDTA (pH 7.4), and embedded in paraffin. The paraffin blocks were sectioned at 5-μm thickness and sectioned joints were performed safranin O staining. The degree of cartilage degradation was scored 0–6 grade using OARSI scoring system and images were acquired by EVOS FL Auto software v.1.7.

### Generation of *Acot12*^*−/−*^ mice

Global *Acot12* knockout mice were generated by germline transmission of an RGEN-induced mutant allele. *Acot12* specific guide RNA and Cas9 protein (each 50 ng/μl) were injected into the cytoplasm of C57BL/6N mouse eggs and transferred into the pseudo-pregnant foster female mice. The absence of *Acot12* in the mutants was confirmed by routine tail DNA genotyping and western blotting. The absence of *Acot12* was confirmed by standard PCR genotyping. Genotyping primers were designed to distinguish *Acot12*^*+/+*^ or *Acot12*^*+/−*^ or *Acot12*^*−/−*^ alleles: forward: 5’-agccaggacgatggagtcga-3’; reverse: 5’-ggtgtccatccacttgagca-3’.

### Transmission electron microscopy

Chondrocytes fixed in 2.5% glutaraldehyde solution were post-fixed with 0.1 M OsO_4_ and embedded with Epoxy Embedding Medium Kit (Sigma-Aldrich). The epoxy embedded block was ultra-microsectioned using EM UC7 (Leica) and stained with uranyl acetate and lead citrate. The microstructure image was analyzed with 120 kV transmission electron microscope (HITACHI).

### Alcian blue staining

Cells were rinsed with PBS and fixed in 4% paraformaldehyde for 20 min. Fixed cells were stained in 1% Alcian blue in 0.1 N HCl for 24 h at room temperature, rinsed with 0.1 N HCl, and captured bright-field image by EVOS FL Auto software v.1.7. Stained Alcian blue was extracted by 6 M guanidine HCl for 2 h and measured absorbance at 600 nm.

### Whole-mount skeletal staining

Postnatal 0 pups were euthanized and removed skin, visceral organ, eye, adipose tissue, and other excess tissue. Specimens were fixed by 95% EtOH for 24 h. Specimens were submerged in alcian blue solution overnight at room temperature and destained in 95% EtOH. To clear the tissue, solution was replaced by 2% KOH solution for 12 h and replaced with Alizarin red solution for 24 h at RT. Stained specimens were replaced by 20% glycerol/1% KOH, 50% glycerol/1% KOH, and 80% glycerol/1% KOH for 24 h each and transferred to 100% glycerol.

### Biochemical analysis

The cellular level of FFA and acetyl-CoA were measured using Free Fatty Acid Assay Kit (Dogen; DG-FFA100) and acetyl-CoA Assay Kit (Biovision; K317-100), respectively according to the manufacturer’s instructions.

### TUNEL assay

TUNEL assay was performed using In Situ Cell Death Detection Kit (Roche) on the deparaffinized cartilage section according to the manufacturer’s instructions. Nuclear was stained with 4’,6-diamidino-2-phenylindole, Dihydrochloride (DAPI). Fluorescence images were acquired using fluorescent microscopy.

### Neutral lipid staining

Fixed cells or cryosection of human and mouse cartilages were stained using BODIPY^493/503^ (Thermo Fisher) for 20 min and mounted with DAPI mounting medium (Vector Laboratories). Fluorescence images were acquired with cartilage images were acquired by EVOS FL Auto software v.1.7.

### Lipid peroxidation staining

Live cells were stained using BODIPY^581/591^ (Invitrogen) probe followed by manufacturer’s recommendation, and fluorescence intensity was quantified by image J 1.51j8.

### Cell apoptosis assay

Cells were harvested and incubated with annexin V and propidium iodide (PI) solution from Muse Annexin V & Dead Cell Kit (Luminex) according to the manufacturer’s instructions. The fluorescence of annexin V and PI were analyzed by Muse Cell Analyzer (Merk Millipore).

### Western blot

ACOT12 KO efficiency was confirmed by western blot using mouse liver. Mouse liver protein were extracted using RIPA buffer (Cell Signaling) with 2 mM PMSF. Thirty micrograms of protein was separated by 10% polyacrylamide gel electrophoresis containing 0.1% SDS and transferred to nitrocellulose membranes (GE Healthcare). The membranes were incubated for 1 h at RT in TBS-T with 5% skim milk and probed with the following primary antibodies: ACOT12 (1:1000 dilution, Mybiosource; #MBS273137), GAPDH (1:10,000 dilution, Bioworld; #AP0066). The blots were developed with a HRP-conjugated anti-rabbit secondary antibody (1:2000 dilution, Bethyl Laboratories) and visualized with SuperSignal West Pico PLUS Chemiluminescent Substrate (Thermo Fisher).

### Immunohistochemistry

For immunohistochemistry, antigen retrieval was performed with deparaffinized-rehydrated cartilage sections using 0.01 M sodium citrate buffer (0.05% Tween 20, pH 6.0). Non-specific antibody-binding sites were blocked by incubation in 2.5% normal horse serum from ImmPRESS Universal Antibody Kit (Vector Laboratories). Sections were incubated overnight with the followed primary antibodies: ACOT12 (1:200 dilution, Mybiosource; #MBS273137,), ADAMTS4 (1:100 dilution, Abcam, #ab28285), FASN (1:100 dilution, Cell Signaling; #3180,), HA-tag (1:100 dilution, Cell Signaling; #3724), MMP13 (1:200 dilution, BioVision; #3533), PPARα (1:100 dilution, Abcam; #ab8934), and SCD1 (1:100 dilution, Abcam; #ab19862). After incubation with peroxidase-conjugated secondary antibody for 30 min, antigen was detected with ImmPACT DAB Substrate (Vector Laboratories). Hematoxylin or methyl-green was used to counterstain.

### Quantitative real-time (qRT)-PCR

Total RNA was isolated from liver tissue using RNAiso Plus (TaKaRa) according to the manufacturer’s instructions. Then, 1 μg RNA was reverse transcribed using the 5× All-In-One RT Master Mix (ABM). Real-time PCR was performed with ABI StepOnePlus instrument (Applied Biosystems) using AMPIGENE qPCR Green Mix Hi-ROX (Enzo). The cycling protocol was 40 cycles of 95 °C for 10 s, 57 °C for 15 s, and 72 °C for 10 s. All qRT-PCR reactions were performed in triplicate. The relative expression level of each gene was normalized to 18 S rRNA expression level. Forward and reverse primer sequences were listed in Supplementary Tables S1 and S2 (used in Fig. 1f), S3 (used in Fig. 3a).

### Synthesis of fluorescein isothiocyanate (FITC)-labeled chitosan (chitosan-FITC)

Chitosan (50 mg, 267.4 μmol of NH2) was dissolved in 1% (v/v) acetic acid solution (2.5 mL). FITC (10.4 mg, 26.7 μmol) in 0.5 ml methanol was slowly added onto the chitosan and reacted for 4 h in the dark. The obtained product was purified by dialysis (MWCO: 12–14 kDa, SpectraPor, USA) against an HCl solution (pH 5) for 2 days, followed by distilled and deionized water (DDW) for 2 days under darkness. The final product was lyophilized and held in the dark.

### Preparation of chitosan-FITC/acetyl-CoA complexes

A chitosan-FITC stock solution was prepared by dissolving chitosan-FITC (1 mg) in 0.05 N HCl solutions (1 ml). A reaction solution was prepared by making a 1:10 dilution of stock solution with pH 7.4 PBS. Ten microliters of acetyl-CoA (50 mM) were added into the reaction solution and reacted for 6 h to ensure the formation of chitosan-FITC/acetyl-CoA complexes. The final product was purified by dialysis (MWCO: 12–14 kDa, SpectraPor, USA) against the DDW for 1 day.

### Ethical approval

All animal studies were approved by the Wonkwang University Animal Care and Use Committee (#WKU18-23, WKU19-09, WKU20-61) and were in compliance with the institutional guidelines. Human cartilage tissue collection was approved by the Human Subjects Committee of Wonkwang University Hospital (WKUH 201605-HRBR-041) and studies were performed in compliance with the institutional guidelines. Written informed consent was obtained from all adult patients or at least one guardian of each patient prior to the start of the experiment.

### Statistical analyses

Results are expressed as the mean ± SD. The mean values for RNA level and biochemical data of two groups were compared by unpaired two-tailed Student’s *t* test. For more than two groups, one- or two-way ANOVA for multiple comparisons were used. Statistical tests with *P* < 0.05 was considered significant and significance was defined as *P < 0.05, **P < 0.01, ***P < 0.001, and ****P < 0.0001. All statistical tests were performed with the software GraphPad Prism 6 (GraphPad).

### Reporting summary

Further information on research design is available in the Nature Research Reporting Summary linked to this article.

## Supplementary information

Supplementary Information

Reporting Summary

## Data Availability

Microarray data of Human OA chondrocytes (GSE16464, GSE64394), primary mouse articular chondrocytes (IL-1β-treated; GSE104793, HIF-2α-overexpressed; GSE104794, ZIP8-overexpressed; GSE104795) for Fig. 1g and OA-induced rat articular chondrocytes (GSE8077) for Fig. 3f were retrieved from GEO database. GSE data was analyzed by QIAGEN Ingeunity Pathway Analysis (IPA) software. Kyoto Encyclopedia of Genes and Genomes (KEGG) pathway analysis was performed by GSEA 3.0. Source data are provided with this paper.

## Source data

Source Data

## Code availability

No unpublished code was used in this manuscript.
