# Supplementary material for: PPARα−ACOT12 axis is responsible for maintaining cartilage homeostasis through modulating de novo lipogenesis
Source: Nat Commun. 2022 Jan 5;13:3. doi: 10.1038/s41467-021-27738-y (PMC8733009; doi:10.1038/s41467-021-27738-y)

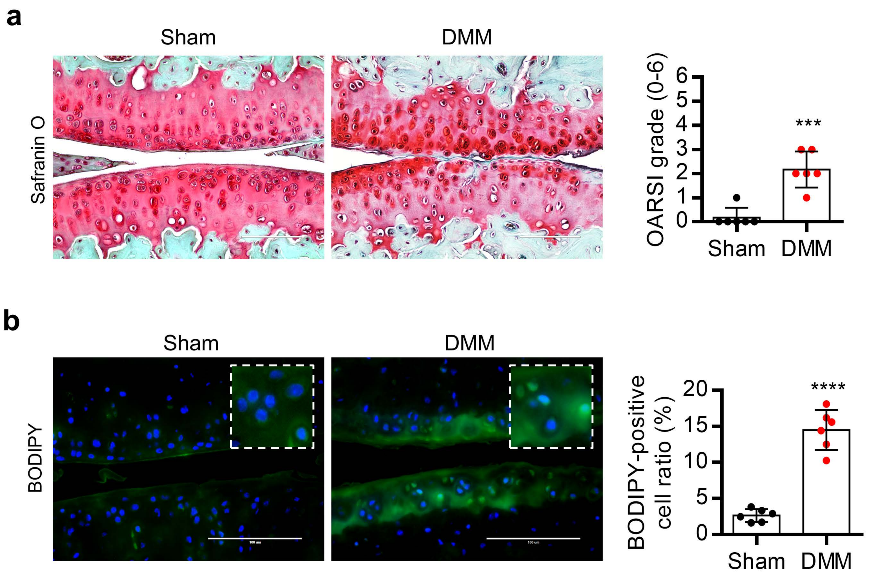

Supplementary Figure 1. Safranin O staining (**a**) and BODIPY$^{483/503}$ staining (**b**) of cartilages from surgery-induced OA models. The degree of cartilage degradation is quantified according to OARSI grade (n = 6), P = 0.0004 and BODIPY-positive cell ratio is indicated by bar-dot plot, $P < 0.0001$. Values are means ± SD. An unpaired Student's t-test (**a, b**) was used for statistical analysis. ***$P < 0.001$, ****$P < 0.0001$.

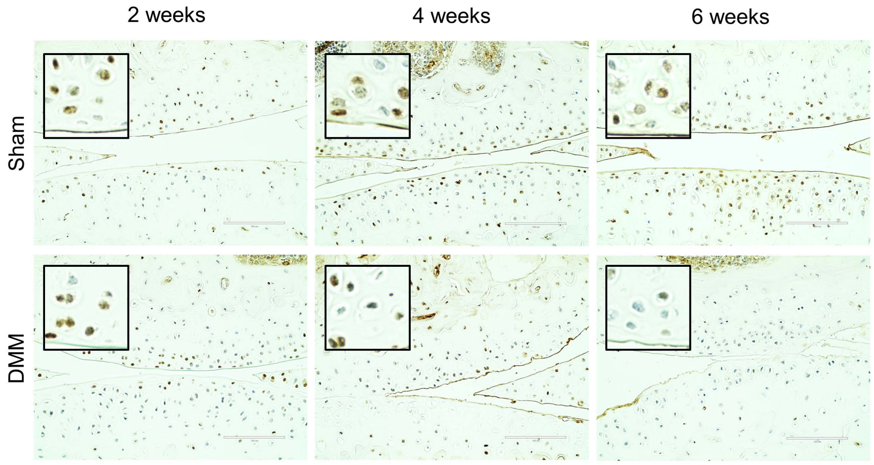

Supplementary Figure 2. PPARα expression in cartilage of OA-induced mice at 2, 4, and 6 weeks of post-DMM surgery (n = 4).

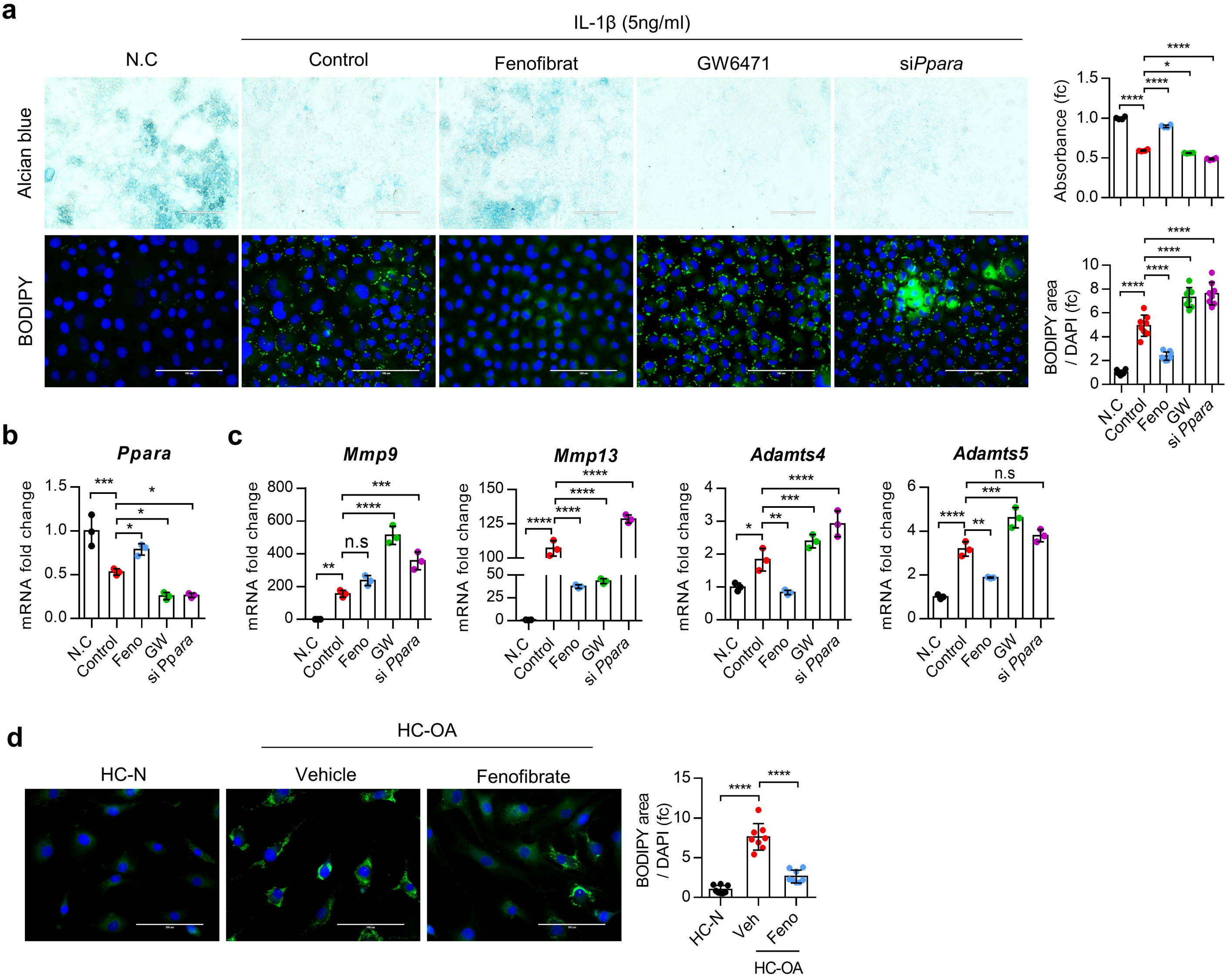

Supplementary Figure 3. a Alcian and BODIPY[483/503] staining of iMACs treated with 100 μM fenofibrate, 10 μM GW6471 or introduced with si*Ppara* in the presence of IL-1β. Extracted Alcian blue staining (*n* = 3) and BODIPY positive cells (n=8) are indicated by bar-dot plot, N.C vs Control, *P* < 0.0001, Control vs Feno, *P* < 0.0001, Control vs GW, *P* = 0.0315, Control vs si *Pa, P* < 0.0001 for Alcian blue staining, *P* < 0.0001 for BODIPY staining. Scale bar, 100 mm. **b** Expression level of *Ppara* in iMACs treated with 100 μM fenofibrate, 10 μM GW6471 or introduced with si*Ppara* in the absence or presence of IL-1β (*n* = 3), N.C vs Control, *P* = 0.0003, Control vs Feno, *P* = 0.0180, Control vs GW, *P* = 0.0122, Control vs si *Pa, P* = 0.0143. **c** Expression level of *Mmp9*, *Mmp13*, *Adamts4* and *-5* in iMACs treated with 100 μM fenofibrate, 10 μM GW6471 or introduced with si*Ppara* in the absence or presence of IL-1β (*n* = 3), N.C vs Control, *P* = 0.0020, Control vs Feno, *P* = 0.0856, Control vs GW, *P* <0.0001, Control vs si *Pa, P* = 0.0003 for *Mmp9*, N.C vs Control, Control vs Feno, Control vs GW, and Control vs si *Pa, P* <0.0001 for *Mmp13*, N.C vs Control, *P* = 0.0091, Control vs Feno, *P* = 0.0028, Control vs GW, *P* = 0.0732, Control vs si *Pa, P* = 0.0014 for *Adamts4*, N.C vs Control, *P* < 0.0001, Control vs Feno, *P* = 0.0017, Control vs GW, *P* = 0.0008, Control vs si *Pa, P* = 0.1397 for *Adamts5*. **d** BODIPY[493/503] staining of human chondrocytes normal (HC-N) and osteoarthritis (HC-OA) treated w/wo 50 μM fenofibrate. BODIPY positive cells are indicated by bar-dot plot (*n* = 8), HC-N vs HC-OA vehicle, *P* <0.0001, HC-OA vehicle vs feno, *P* = 0.0204. Scale bar, 100 mm. Values are means ± SD. One-way ANOVA followed by Tukey's multiple comparisons test (**a – d**) was used for statistical analysis. n.s, not significant *P* >= 0.05; *$P < 0.05$; **$P < 0.01$; ***$P < 0.001$; ****$P < 0.0001$.

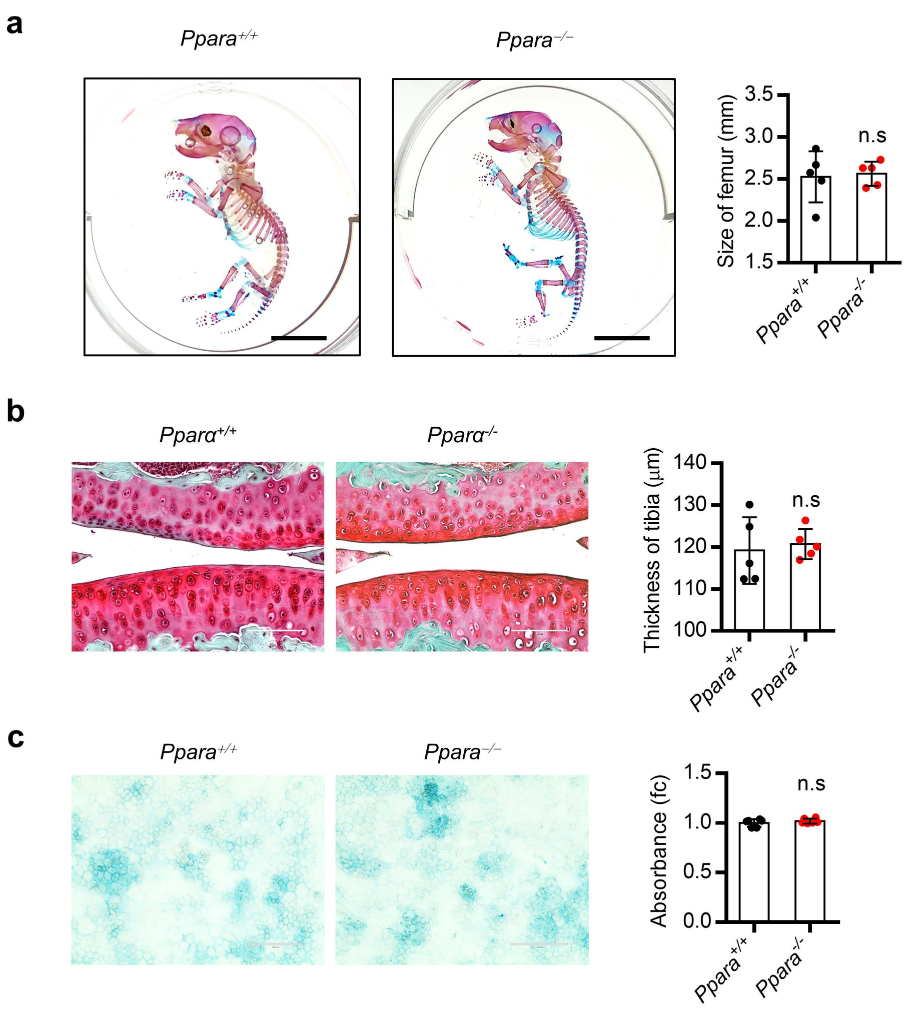

Supplementary Figure 4. Cartilage degradation in *Ppara* knock-out (KO) mice **a** Alizarin red/Alcian blue staining of a newborn skeleton. The size of femur is indicated by bar-dot plot (*n* = 5), *P* = 0.8106. **b** Safranin O staining of *Ppara*$^{+/+}$ and *Ppara*$^{-/-}$ cartilages. The thickness of tibia is indicated by bar-dot plot (*n* = 5), *P* = 0.7065. **c** Alcian O staining of *Ppara*$^{+/+}$ and *Ppara*$^{-/-}$ iMACs. Extracted Alcian blue staining is indicated by bar-dot plot (*n* = 4), *P* = 0.3428. Values are means ± SD. An unpaired Student's t-test (**a – c**) was used for statistical analysis. n.s, non-significant *P* >= 0.05.

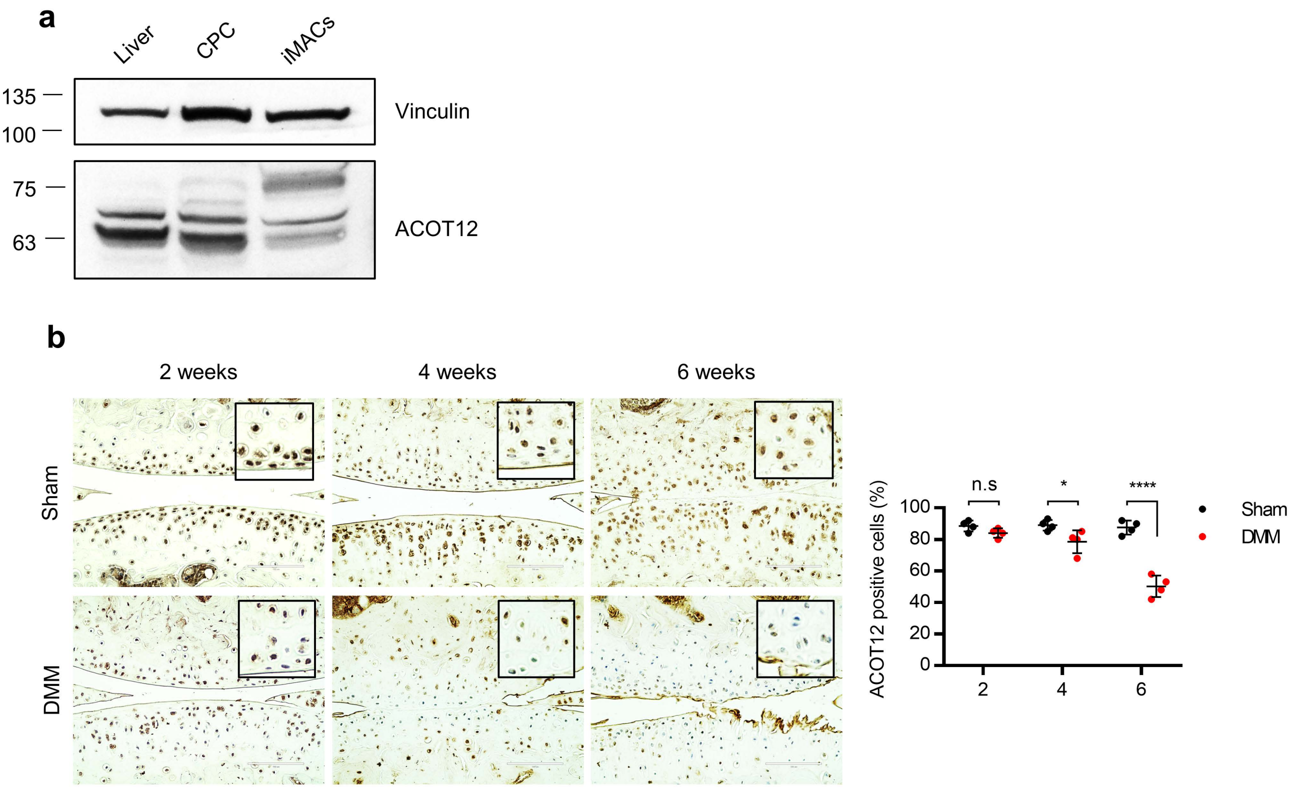

Supplementary Figure 5. a Expression level of ACOT12 (n = 3) in chondrogenic progenitor cell (CPC), immature murine articular chondrocytes (iMACs), and liver. **b** Immunoblotting of ACOT12 in cartilage section of sham- or DMM-induced mice at 2 weeks, 4 weeks, and 6 weeks. Scale bar, 100 mm. ACOT12-positive cells are indicated by bar-dot plot (n = 4), Sham vs DMM at 2 weeks, P = 0.2220, at 4 weeks, P = 0.0085, at 6 weeks, P < 0.0001. Values are means ± SD. Multiple t test followed by Holm-Sidak's method (**b**) was used for statistical analysis. n.s, not significant P >= 0.05; *P < 0.05; ****P < 0.0001.

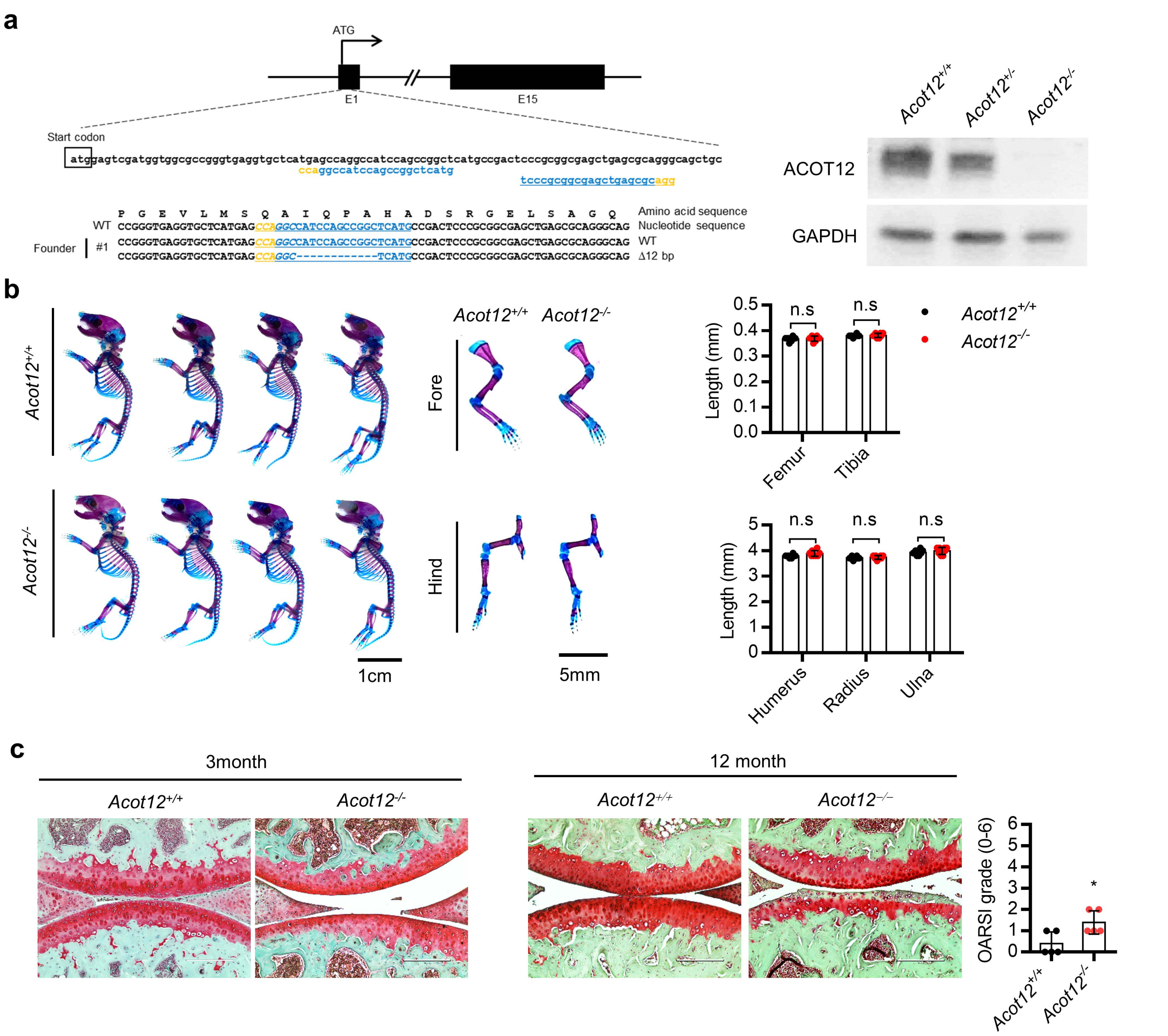

Supplementary Figure 6. Generation of *Acot12* knock-out (KO) mice **a** Target region (blue characters) and protospacer adjacent motif (yellow characters) of *Acot12* RGEN. DNA sequence of the *Acot12* locus from live F0 mice (#1). −, deleted nucleotides (left panel). ACOT12 knockout efficiency was confirmed by western blotting (right panel) (n = 3). **b** An analysis of bone development in *Acot12*$^{-/-}$ mice (n = 4). Alizarin red/Alcian blue staining of a newborn skeleton and fore- and hind-limb. The length of femur and tibia, femur, P = 0.7942, tibia, P = 0.7266 and the length of humerus, radius, and ulna, humerus, P = 0.0465, radius, P = 0.4833, ulna, P = 0.3177 are indicated by bar-dot plot. Scale bar, 1 cm. **c** Safranin O staining in cartilages of 3- and 12-month-old *Acot12*$^{+/+}$ or *Acot12*$^{-/-}$ (n = 5). The degree of cartilage degradation is quantified according to OARSI grade, P = 0.0497. Values are means ± SD. An unpaired Student's *t*-test (**b**) or multiple t test followed by Holm-Sidak's method (**c**) were used for statistical analysis. n.s, not significant $P >= 0.05$; *$P < 0.05$.

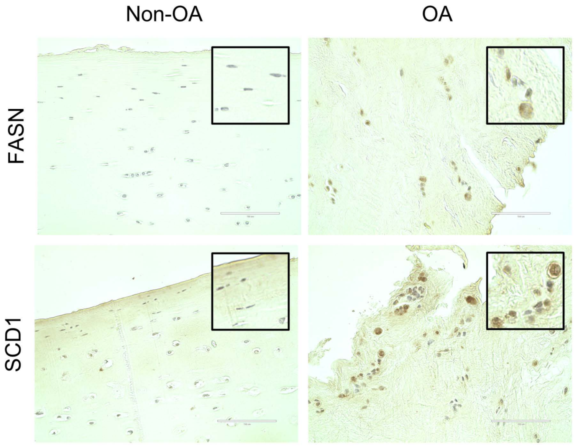

Supplementary Figure 7. Immunohistochemistry of FASN and SCD1 in non-OA (*n* = 4) and OA (*n* = 4) cartilage section of OA patients. Scale bars, 100 mm.

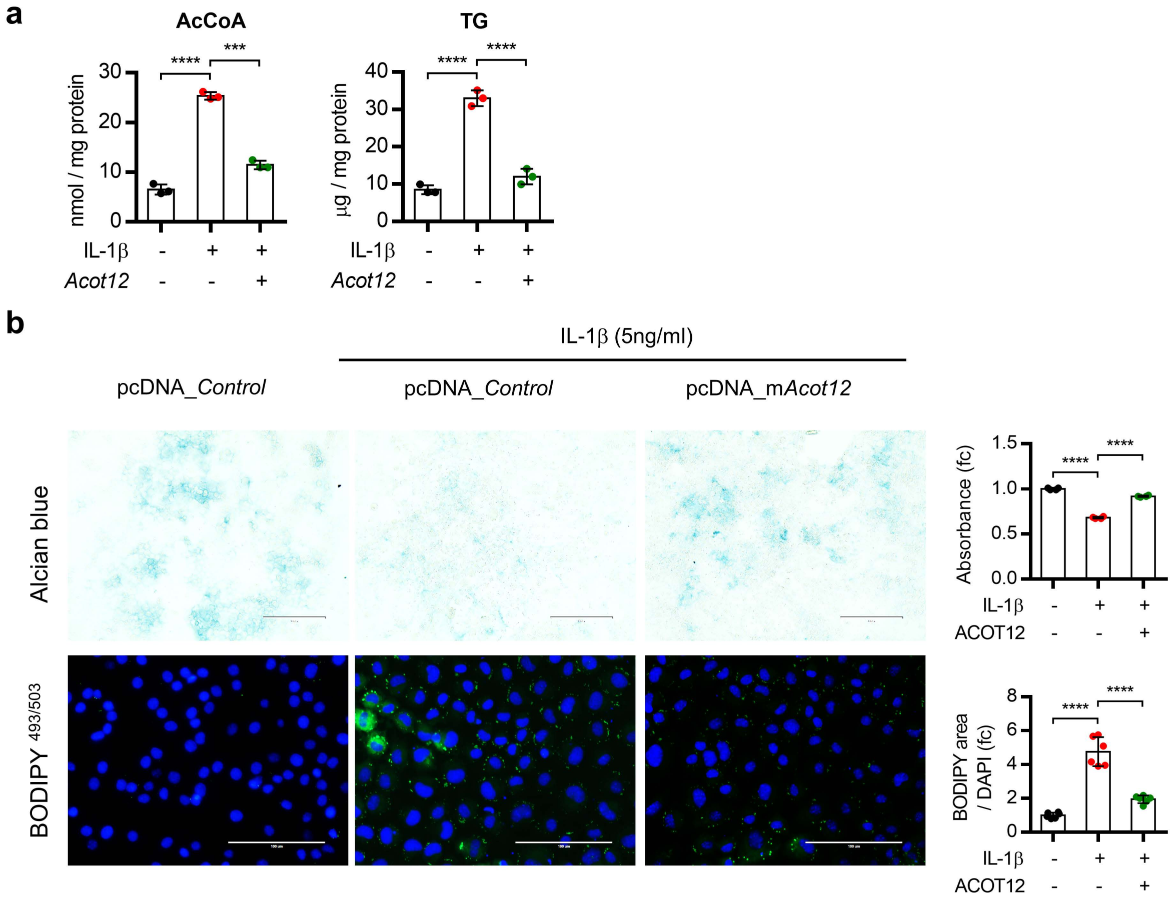

Supplementary Figure 8. ACOT12 (pcDNA_m*Acot12*) was introduced into WT iMACs treated w/wo IL-1β. **a** Cellular level of acetyl CoA (n = 3) Control vs IL-β, P < 0.0001, IL-b vs IL-b + ACOT12, P = 0.0011 and TG (n = 3), Control vs IL-β and IL-β vs IL-β + ACOT12, P < 0.0001. **b** Alcian blue (n = 4) and BODIPY (n = 6) staining and the positive cell counting, Control vs IL-β and IL-b vs IL-β + ACOT12, P < 0.0001 for Alcian blue staining, Control vs IL-β and IL-β vs IL-β + ACOT12, P < 0.0001 for BODIPY staining. Values are means ± SD. One-way ANOVA followed by Tukey's multiple comparisons test (**a, b**) was used for statistical analysis. ***P < 0.001; ****P < 0.0001.

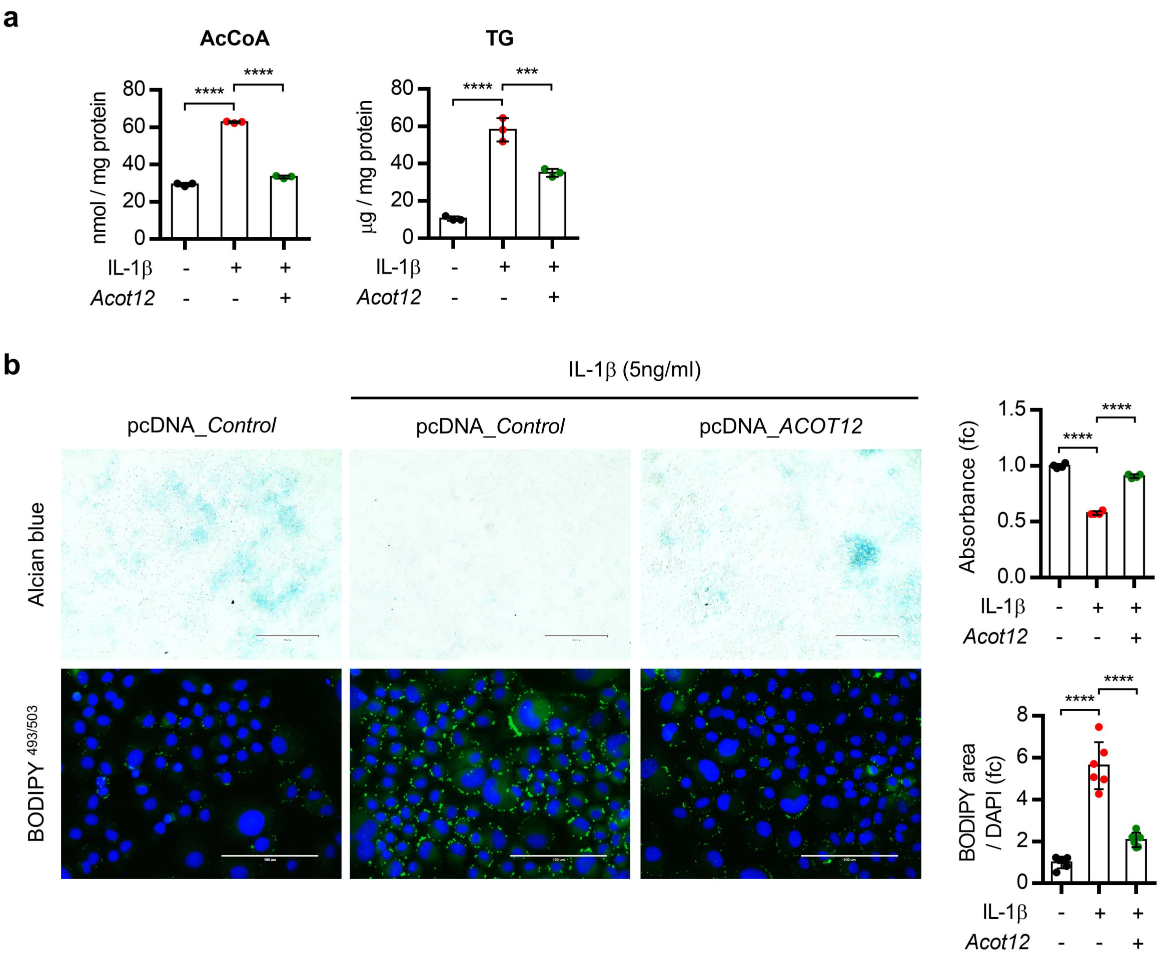

Supplementary Figure 9. ACOT12 (pcDNA_mAcot12) was introduced into *Acot12*$^{-/-}$ iMACs treated w/wo IL-1β. Scale bars, 100 mm. **a** Cellular level of acetyl CoA (n = 3), Control vs IL-β and IL-β vs IL-β + ACOT12, P < 0.0001 and TG (n = 3), Control vs IL-β P < 0.0001, IL-β vs IL-β + ACOT12, P = 0.0008). **b** Alcian blue (n = 4) and BODIPY (n = 6) staining and the positive cell counting, Control vs IL-β and IL-β vs IL-β + ACOT12, P < 0.0001 for Alcian blue staining, Control vs IL-β and IL-β vs IL-β + ACOT12, P < 0.0001 for BODIPY staining. Values are means ± SD. One-way ANOVA followed by Tukey's multiple comparisons test (**a, b**) was used for statistical analysis. ***P < 0.001; ****P < 0.0001.

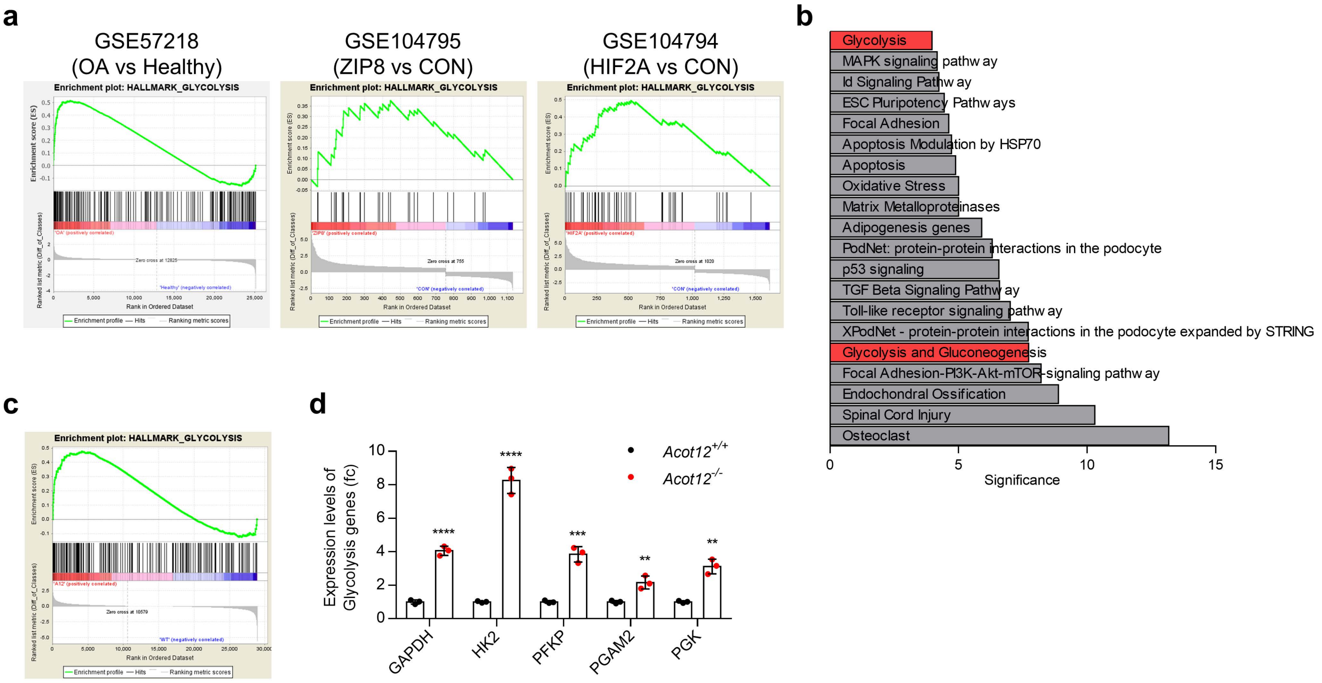

Supplementary Figure 10. a Gene set enrichment analysis (GSEA) was perfomed analysis using GSE57218, GSE104794 and GSE104795. **b-c** Enrichment plot of KEGG analysis and GSEA from RNA sequencing data of OA patient (OA vs non-OA). **d** Expression level of glycolysis genes in human OA chondrocyte versus non-OA chondrocyte (n = 3), GAPDH, $P < 0.0001$, HK2, $P < 0.0001$, PFKP, $P = 0.0005$, PGK, $P = 0.0012$, PGAM2, $P = 0.0071$. Data are means ± SD. Multiple *t*-test followed by Holm-Sidak's method (**d**) was used for statistical analysis. **$P < 0.01$; ***$P < 0.001$; ****$P < 0.0001$..

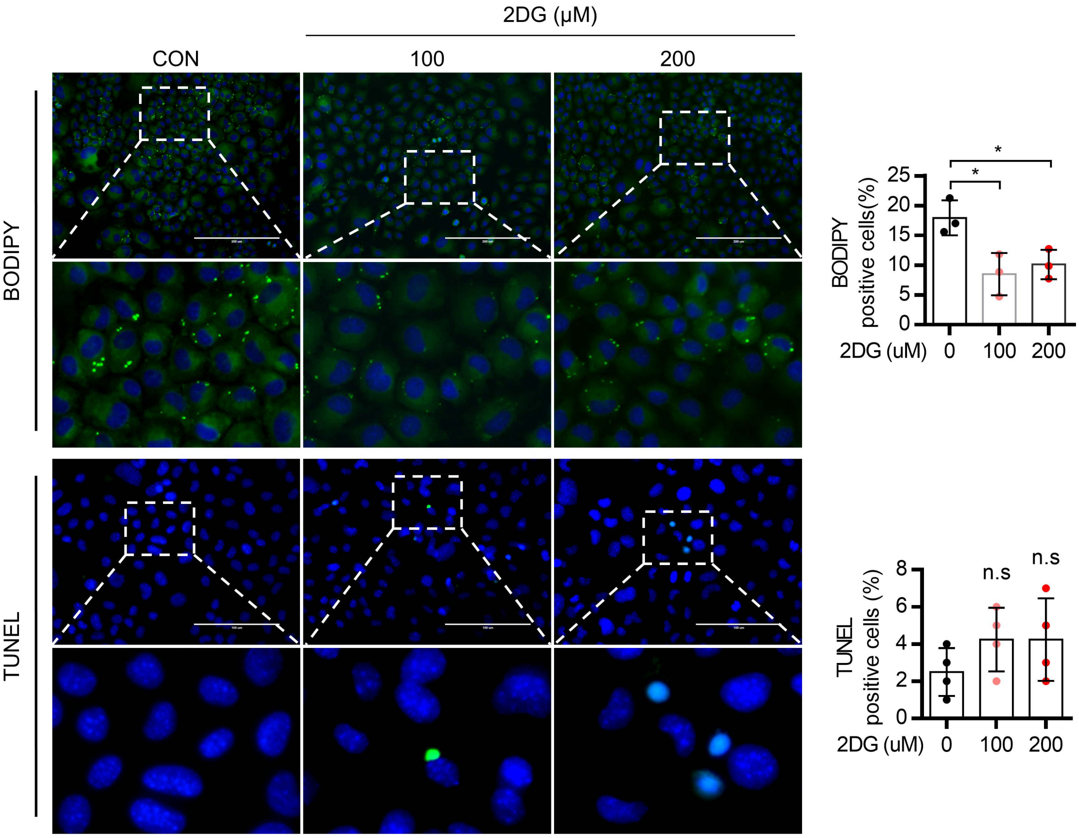

Supplementary Figure 11. Glycolysis inhibition induces cell death. BODIPY (*n* = 3) and TUNEL (n=4) staining of iMACs treated with glycolysis inhibitor, 2DG and BODIPY, and TUNEL-positive cells were indicated by bar-dot graph. 0 vs 100 μM 2DG, *P* = 0.0153, 0 vs 200 μM 2DG, *P* = 0.0338 for BODIPY staining, 0 vs 100 μM 2DG, *P* = 0.3224, 0 vs 200 μM 2DG, *P* = 0.3224 for Tunnel staining, Scale bar, 200 mm, upper; 100 mm, bottom. Data are means ± SD. One-way ANOVA followed by Dunnett's multiple comparisons test was used for statistical analysis. n.s, not significant *P*> = 0.05; **P* < 0.05.

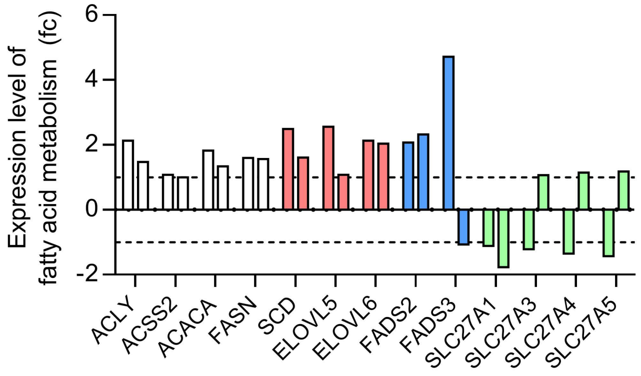

Supplementary Figure 12. Expression level of fatty acid metabolism related genes in human OA chondrocyte versus non-OA chondrocyte.

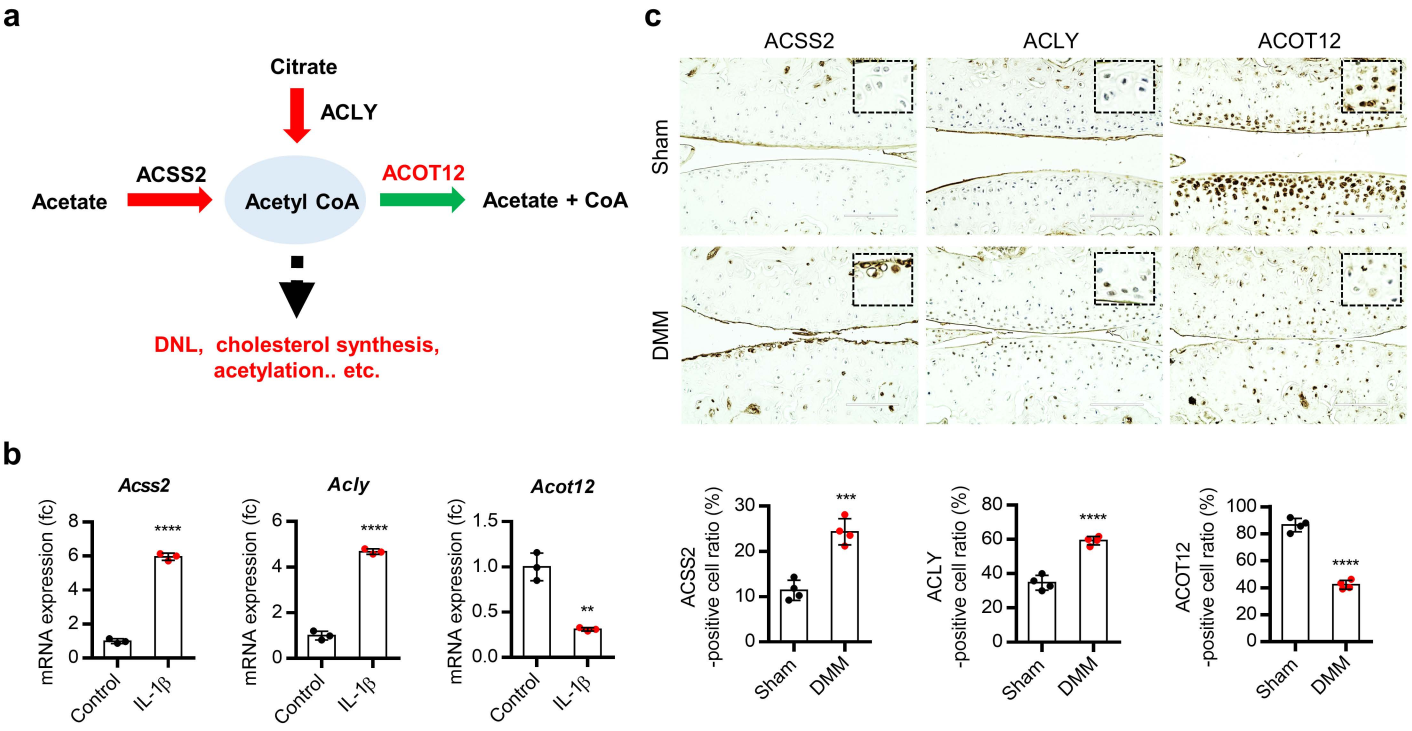

Supplementary Figure 13. ACOT12 is involved in lipid metabolism during OA pathogenesis. **a** Schematic diagram of Acetyl-CoA metabolism. Red arrow indicates synthetic reaction and green arrow indicates degradative reaction. **b** Transcription levels of *Acss2* ($P < 0.0001$), *Acly* ($P < 0.0001$), and *Acot12* ($P = 0.0015$) transcript in WT iMACs treated with 5 ng / ml IL-1β ($n = 3$). **c** Immunohistochemistry and positive cell counting of ACSS2 ($P = 0.0004$), ACLY ($P < 0.0001$), and ACOT12 ($P < 0.0001$) in Sham and DMM-induced mouse cartilage ($n = 4$). Scale bars, 100 mm. Values are means ± SD. An unpaired Student's *t*-test (**b, c**) was used for statistical analysis. **$P < 0.01$, ***$P < 0.001$, ****$P < 0.0001$.

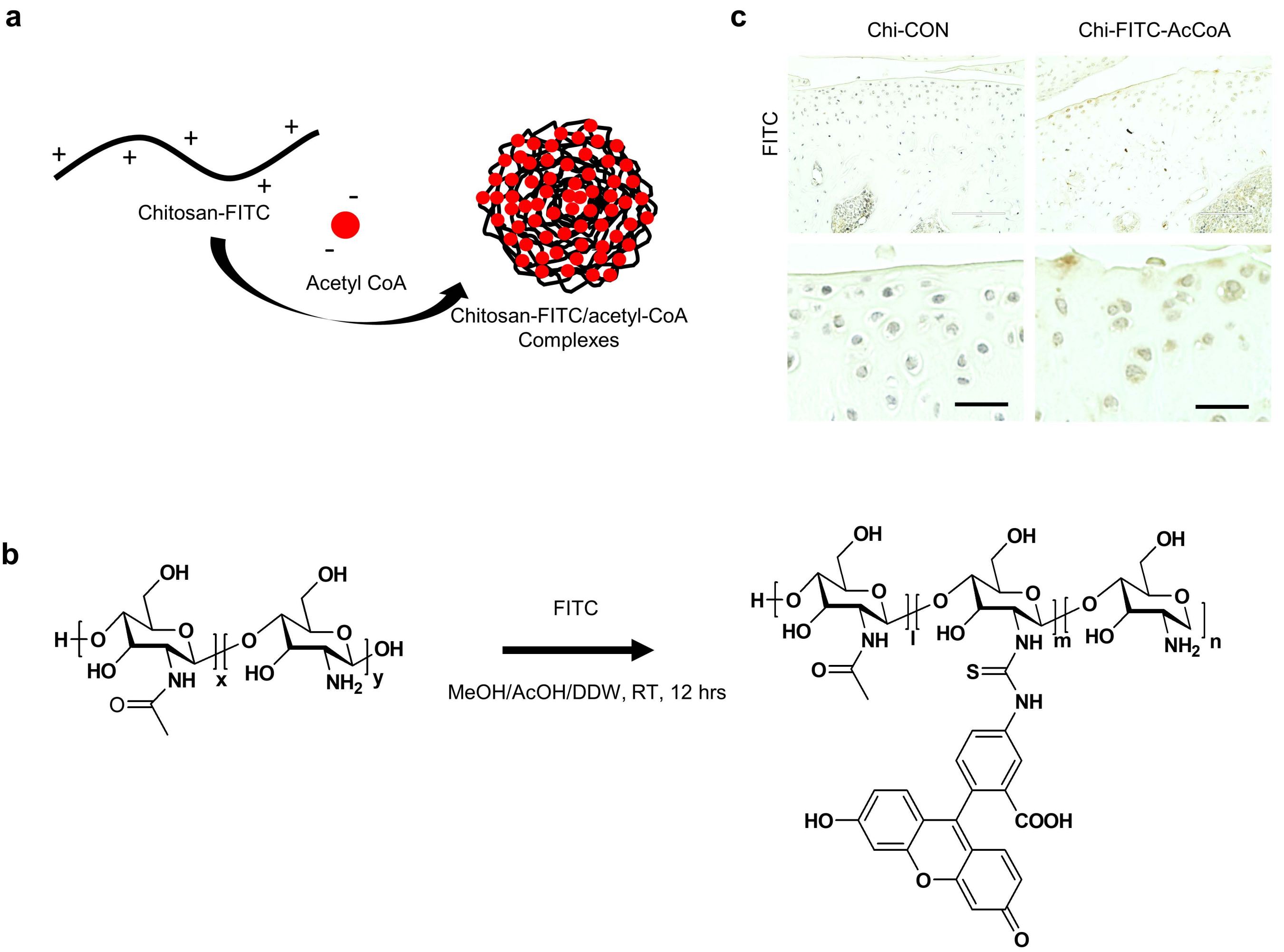

Supplementary Figure 14. Formation and delivery of acetyl CoA into mouse cartilage. **a** Schematic diagram of the formation of chitosan-FITC-Acetyl CoA. **b** Synthesis and chemical structures of chitosan-FITC. **c** Immunohistochemistry of Fluorescence in mouse cartilage injected chitosan or chitosan-FITC (chitosan-FITC; *n* = 4). Scale bar, 100 mm in upper; 25 mm in bottom.

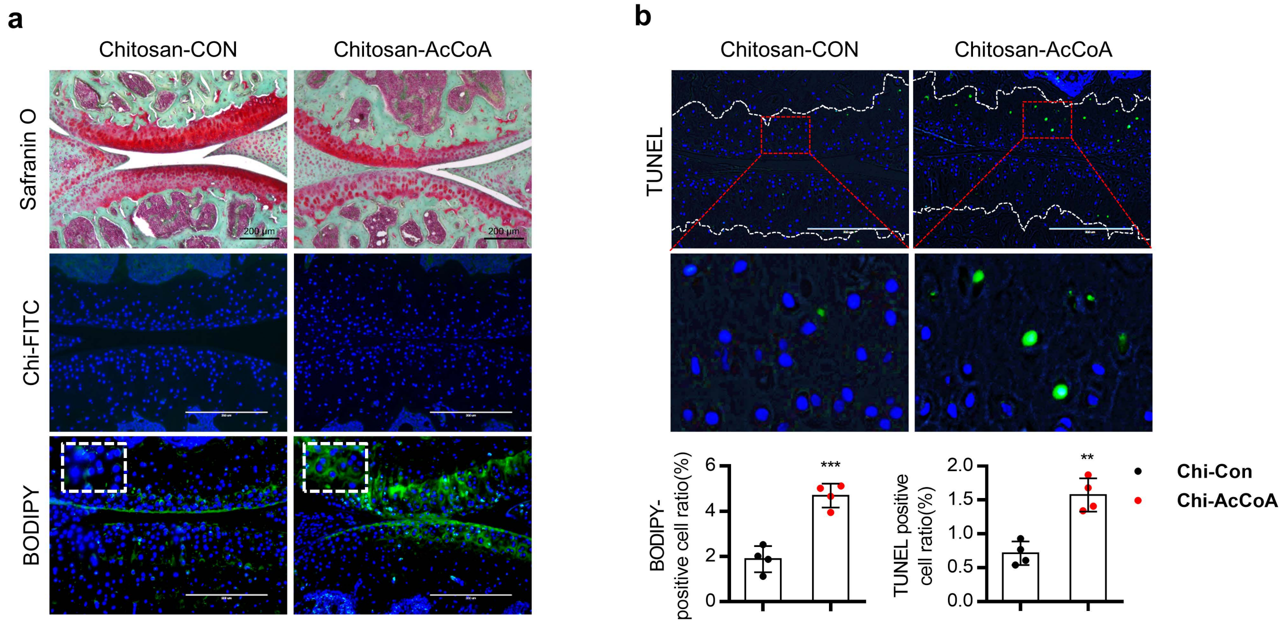

Supplementary Figure 15. Cartilage degradation by injection of Chitosan-FITC conjugated AcCoA. Safranin O, FITC, BODIPY, and TUNEL analysis (*n* = 4) in sham operated mouse cartilage injected chitosan or chitosan-AcCoA. BODIPY (*P* = 0.0004) and TUNEL-positive cells (*P* = 0.0012) are indicated by bar-dot plot. Scale bar, 200 μm. Values are means ± SD. An unpaired Student's *t*-test was used for statistical analysis. ***P* < 0.01; ****P* < 0.001.

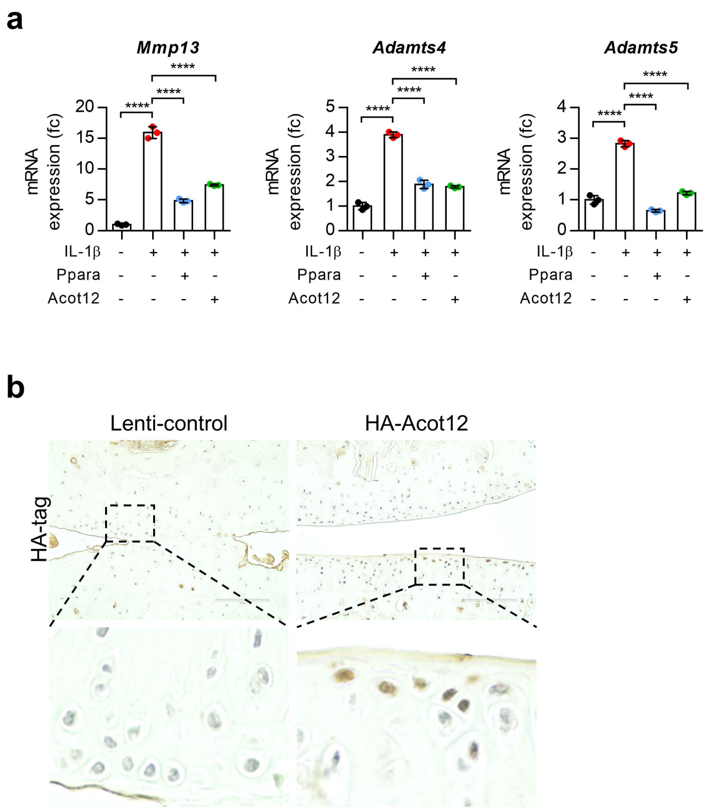

Supplementary Figure 16. a Transcription level of *Mmp13* (P < 0.0001), *Adamts4* (P < 0.0001) *and Adamts5* (P < 0.0001) in *Ppara*$^{-/-}$ iMACs introduced with PPARα or ACOT12 expression vector in the absence or presence of IL-1b (n = 3). **b** Immunohistochemistry of HA in cartilage of *Ppara*$^{-/-}$ mice introduced with lentivirus containing HA-tagged Acot12 (n = 4). Scale bars, 100 mm. Values are means ± SD. One-way ANOVA was used for statistical analysis. ***P < 0.001; ****P < 0.0001.

Supplementary Table 1. Primer list used in this study.

Symbol	Forward	Reverse
Human *MMP13*	ctgcatatgagcacccttct	ttttggaagacccagttcag
Human *ADAMTS4*	cgctttgcttcactgagtagat	ctgttagcaggtagcgctttag
Human *ADAMTS5*	gcactggctactatgtggtatt	agccagttctcacacacttc
Human *ACOT12*	gtccagcacatttggcttatc	catcttcactcacccagtctatg
Human *PPARA*	cctgcaagaaatgggaaacatc	gccaggacagcttcctaaat
Human *18S rRNA*	ctgagaaacggctaccacatc	gcctcgaaagagtcctgtattg
Mouse *Acot12*	ccaccaccttggagaagataaa	gtgaggtcagacaagagatgataag
Mouse *Ppara*	cggtgtgtatgaagccatct	taaggaactcgcgtgtgataaa
Mouse *Acan*	gagacttctgcctctggaatag	ctccagaaggaatcccactaac
Mouse *Col2a1*	ctggtttggagagaccatgaa	gaggaaagtcatctggacgttag
Mouse *Mmp9*	tctgtatggtcgtggctctaa	ggaggtatagtgggacacatagt
Mouse *Mmp13*	ccctgatgtttcccatctatacc	ttcatcgcctggaccataaag
Mouse *Adamts4*	gcattccatggtacagggtta	agttgacagggtttcggatg
Mouse *Adamts5*	tgccacagacccaactaaag	ccatggctgatgacagagtt
Mouse *Acaca*	acattccgagcaagggataag	gggatggcagtaaggtcaaa
Mouse *Fasn*	agacccgaactccaagttattc	gcagctccttgtatacttctcc
Mouse *Scd1*	caacttcaccacgttcttcatc	cccgtctccagttctcttaatc
Mouse *Acss2*	caccttctggcaaacagaaac	ctacaccgaagaatgggaaaga
Mouse *Acly*	gagcatgagcagtgaagaca	catggcggcatacagtagatag
Mouse *Rn18s*	ccagtaagtgcgggtcataag	ggcctcactaaaccatccaa

Supplementary Table 2. Primer list used in Fig. 1f.

Symbol	Human		Mouse	
	Forward	Reverse	Forward	Reverse
ACACA	gcaggtcacacgtctctttat	ccagcctgtcatcctcaatatc	agccagaagggacagtagaa	ctcagccaagcggatgtaaa
ACADM	gagaagaagggtgacgagtatg	ggctttactagcgggtacttta	gagaagaagggtgacgagtatg	ggctttactagcgggtacttta
ACADS	agctccaggtcatccagttc	ctgcctccttgatgaaaggc	cccacagctcaggttaagaa	gatggagtaggccaggtaatc
ACADVL	catgaaggaacctggagtagag	ccacaaacagccgaagaatg	ctttgcagggactcaaggaa	caagcgagcatactgggtatta
ACAT1	tggaggagctctttctctgg	gcaaatactggcaagaccgt	catgatggcctctcaaagtct	tgttgctcctctgctcattac
ACOT1	ggtgaccaaagatggctatg	tgacctaccaggaacaggaa	cgtcatggctctggcttatta	caggtagttcacggcttctt
ACOT2	cttggtgggcagtcctattatc	cccaagtgtttgtggaagaaag	cgtgatggctctggcttatta	caggtagttcacggcttctt
ACOT4	ccacgttggctctagcttatt	gcatgtagcatacggcttct	tacaggctcatggaaaggaaag	tgggcacatgggaaagtaag
ACOT8	gagaccctcattgaccagtattt	tgggtttactggcttgatctc	actggagcccaaacagatg	aggaaggcgtagtcagagata
ACSS2	ggttctgctactttcccattct	ggctgcttgaacaccagata	caccttctggcaaacagaaac	ctacaccgaagaatgggaaaga
ALDH3A2	catgctggatgaggcctatatt	agtggctgaatggtgagaac	ggcttctcctgactatgaaagg	catccatctctccaccgaaag
APOA	cctgacacaatgctcagacg	cctttgctcagttggtgctt	ctctgggttcaaccgttagtc	tctcctgtctcacccaatct
APOB	ccctcagtcctctccagataaa	gctgcctcttcttcccaatta	aggcttgtcacccttctttc	gccttgtgagcaccagtatta
CD36	cattggtgatgagaaggcaaac	caccacaccaacactgagtaa	ctgggaccattggtgatgaaa	caccactccaatcccaagtaag
CHREBP	ggaagaatttcaaaggcctcaag	ctcttcctccgcttcacatac	cagctgcgggatgaaataga	caaagcgctgatgtgtgatg
CIDEA	gtgaaggccaccatgtatga	tgtgcccagatagatgagaaac	gcaaccaaagaaatcgggaatag	ctcgtacatcgtggctttga
CIDEC	gattgatgtggcccgtgtaa	agcagtgcagatcataggaaag	gctgaaccctcaggactttatt	cttgtagcagtgcaggtcatag
CPT1A	tcctggtgggctacaaattac	acagcagatccatggcataata	gaagtgtcggcagacctattt	gtcctcctctctatatccctgtt
CPT1B	gctgctaagaagcaccagaata	ggagaccaagtaaaggcagaag	attctgtgcggcccttatt	tgacttgagcaccaggtattt
CPT2	cgagctgactgatgccttaaa	ttctttgcctcctctctgaaac	ctcagaaatccaggcacatcta	ctcggttctcactggtcaaata
CRAT	cttcaaggcacaccaggat	actcctcctcactcacgat	ggaggcccatatcaacttctc	atgtccagcagagctttctc
CROT	tggctaaatcaactgaagaacga	cttcaagtgaaggaacaggca	tgctgtgatcatgctcct	ctgaacccttccatcttcctt
DGAT1	aactaccgtggcatcctgaa	agagaaaccacctggatggg	ggccttactggttgagtctatc	gttgacatcccggtaggaataa
EHHADH	aggaggaggagctgtttctat	ggagggagttgaccacttattt	gtggagcaaatgacaacttctg	ggcttctggtatcgctgtatt
ELOVL5	acttcttctgtcagggcaca	ccgtgatctggtggttgttc	ggtgtgtgggaaggcaaatac	tggagaagtagtaccaccagag
ELOVL6	tctgctctgtatgctgcctt	agactgcaagggtcagagac	ccgaactaggtgacacgatatt	tgtaggagtaccaggagtacag
FABP4	gaagtaggagtgggctttgc	ttcctggcccagtatgaagg	ggatggaaagtcgaccacaata	tggctcatgccctttcataa
FASN	ctaggtttgatgcctccttctt	gatggcttcataggtgacttcc	agacccgaactccaagttattc	gcagctccttgtatacttctcc
HACL	atgctggtactttcggaaca	gaaaacccaaatgcactgtc	tttggactgcctccaagatac	tcacagaaggcctgacattatt
HAO1	aggcagagaagatgggctacaa	ttttcatcctgagttgtggcgg	tcttgggctacctcctcaata	cttctcagctcgcttcactatc
LDLR	ctcccgccaagatcaagaaa	gtttggagtcaacccagtaga	atccaccgcaacatctactg	ggaacagtgtcctcctctttac
MOGAT1	gacctgtttcctggctttact	cagcccaacactcatcacata	gacctcagagcagattgaagag	gtgctccggaatcccatatt
PHYH	gctttcggaatgagtttgaa	tggaccttcgtgatcatctt	ctccagatgttggcaagaaga	gctgtccaagcacaaacaatta
PPARA	cctgcaagaaatgggaaacatc	gccaggacagcttcctaaat	cggtgtgtatgaagccatct	taaggaactcgcgtgtgataaa
PPARG	cccaagtttgagtttgctgtg	gcggtctccactgagaataatg	ctggcctccctgatgaataaag	aggctccataaagtcaccaaag
SCD1	cctgcagaatggaggagataag	gccttccttatccttgtaggtg	caacttcaccacgttcttcatc	cccgtctccagttctcttaatc
SLC27A2	ctcttgccttgcggactaaa	ccgaagcagttcaccgatatac	ctggacaaagtagacggagtg	cctgtggttcccgaagtataaa
SREBF1	gtgacttccctggcctattt	cttcaagagaggagctcaatg	acttccctggcctatttgacc	ggcatggacgggtacatctt
SRIT6	cagtcttccagtgtggtgtt	ctcaaaggtggtgtcgaact	cgtctggtcattgtcaacct	gagtctgcacatcacctcatc
VLDLR	caggaagattggcttagagagg	gcttagatcggcccagaatag	gccgagtctgatcttcactaatc	ccgtgttccttagttgctctac
18s rRNA	ctgagaaacggctaccacatc	gcctcgaaagagtcctgtattg	ccagtaagtgcgggtcataag	ggcctcactaaaccatccaa

Supplementary Table 3. Primer list used in Fig. 3a.

Symbol	Forward	Reverse
Abca1	gtttccgggaagtgtcctaaa	ctgggagaggatgctgaatatc
Abcb4	atggctggatcagtgctctt	gagaatgggttcctgggaca
Abcd2	gaccatgcctatgagacctattt	gtaagagactggtcagggtttg
Abcd3	cttgcttgtttctgggctattc	cgtatctgtattctccttcgtactt
Acaa1a	catcccagagactgtacctttg	gccaatgtcataagacccatttc
Acaa2	gacttctctgccaccgattta	ttgcccacgatgacactatc
Acacb1	gttccgtaatgaacgtgcca	acgtactgatcggccatctt
Acadm	gagaagaagggtgacgagtatg	ggctttactagcgggtacttta
Acads	cccacagctcaggttaagaa	gatggagtaggccaggtaatc
Acadvl	ctttgcagggactcaaggaa	caagcgagcatactgggtatta
Acat1	catgatggcctctcaaagtct	tgttgctcctctgctcattac
Acot12	ctcagtgcacagtgggatataag	cttctccaaggtggtggtaatc
Acox1	cgcacatcttggatggtagt	ggcttcgagtgaggaagttatag
Acsl1	gcttgtggatgtggaagaaatg	tcttgctgggtctttcaagtag
Acsl3	ggctgtgcactggagatatt	gtactctcctgcctgtagtttc
Agpat2	cagaagaaactggaggtggatg	cacagcgcttagggagtattt
Aldh9a1	gaggttctagagcgagctaatg	ctccactgggctgacattatag
Apoa1	ctctgggttcaaccgttagtc	tctcctgtctcacccaatct
Cidec	gctgaaccctcaggactttatt	cttgtagcagtgcaggtcatag
Cpt1a	gaagtgtcggcagacctattt	gtcctcctctctatatccctgtt
Cpt2	ctcagaaatccaggcacatcta	ctcggttctcactggtcaaata
Cyp4a10	ccctgatggacgctctttac	gggtcaaacacctctggatt
Cyp7a1	caccttgaggatggttcctataa	tcaaagggtctgggtagatttc
Ech1	cgcgatgacagtttccagta	cagagatcgaaggctgatgtt
Elovl6	ccgaactaggtgacacgatatt	tgtaggagtaccaggagtacag
Fabp3	aggcaaactcatccatgtgc	tgccatgagtgagagtcagg
Fads1	tggctcaccctttggatctt	gtcatgctgtagccaacctg
Hadha	agacatcggagctgtctttg	cactaccttctgagcaccatac
Hmgcs2	agaccaaggcctccctttac	attcttgggcagagtggtga
Lepr	aatgcaactgcagtcttcgg	cacaccggcagatgtgattt
Pctp	gatgtggacgggaggaagat	gccaggctctgtttgtactg
Plin2	ctcaggaggagctggagatg	cgagagcagagcttggtaga
Slc22a5	ataatcctgtggctgaccatatc	ccagtaggaagcagttcacatag
Slc25a20	gcagacacaaccaccaagtt	cctgtgatgccctctctcat
Slc27a2	ctggacaaagtagacggagtg	cctgtggttcccgaagtataaa
Slc27a4	ggctctcatcaacaccaacc	tagctgaggccatctcactg
Vldlr	gccgagtctgatcttcactaatc	ccgtgttccttagttgctctac

Uncropped image of Supplementary Fig. 5a

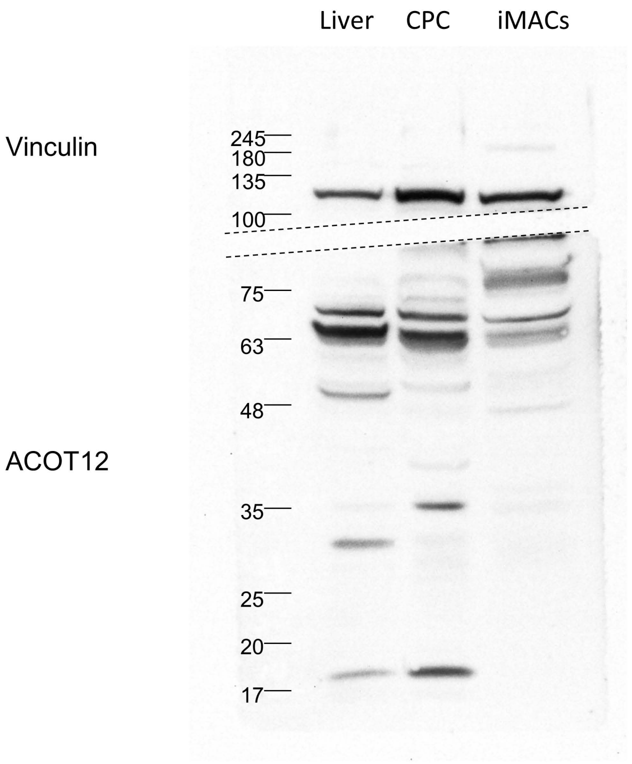

Uncropped image of
Supplementary Fig. 6a

ACOT12

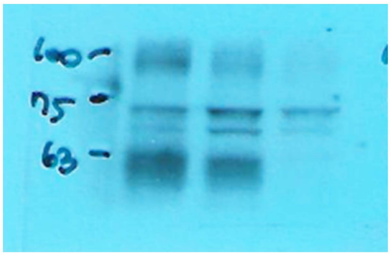

GAPDH

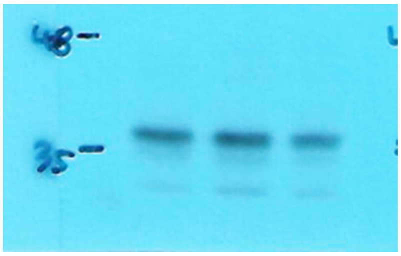

Supplement: Supplementary file 1 — Supplementary Information [file 41467_2021_27738_MOESM1_ESM.pdf]
