# Supplementary material for: PPARα−ACOT12 axis is responsible for maintaining cartilage homeostasis through modulating de novo lipogenesis
Source: Nat Commun. 2022 Jan 5;13:3. doi: 10.1038/s41467-021-27738-y (PMC8733009; doi:10.1038/s41467-021-27738-y)
Supplement: Supplementary file 2 — Reporting Summary [file 41467_2021_27738_MOESM2_ESM.pdf]

nature portfolio

Corresponding author(s): Eun-Jung Jin

Last updated by author(s): 2021 11 30

Reporting Summary

Nature Portfolio wishes to improve the reproducibility of the work that we publish. This form provides structure for consistency and transparency in reporting. For further information on Nature Portfolio policies, see our Editorial Policies and the Editorial Policy Checklist.

Statistics

For all statistical analyses, confirm that the following items are present in the figure legend, table legend, main text, or Methods section.

n/a	Confirmed	
☐	☒	The exact sample size (*n*) for each experimental group/condition, given as a discrete number and unit of measurement
☐	☒	A statement on whether measurements were taken from distinct samples or whether the same sample was measured repeatedly
☐	☒	The statistical test(s) used AND whether they are one- or two-sided<br>*Only common tests should be described solely by name; describe more complex techniques in the Methods section.*
☐	☒	A description of all covariates tested
☐	☒	A description of any assumptions or corrections, such as tests of normality and adjustment for multiple comparisons
☐	☒	A full description of the statistical parameters including central tendency (e.g. means) or other basic estimates (e.g. regression coefficient) AND variation (e.g. standard deviation) or associated estimates of uncertainty (e.g. confidence intervals)
☐	☒	For null hypothesis testing, the test statistic (e.g. *F*, *t*, *r*) with confidence intervals, effect sizes, degrees of freedom and *P* value noted<br>*Give P values as exact values whenever suitable.*
☒	☐	For Bayesian analysis, information on the choice of priors and Markov chain Monte Carlo settings
☒	☐	For hierarchical and complex designs, identification of the appropriate level for tests and full reporting of outcomes
☒	☐	Estimates of effect sizes (e.g. Cohen's *d*, Pearson's *r*), indicating how they were calculated

Our web collection on statistics for biologists contains articles on many of the points above.

Software and code

Policy information about availability of computer code

Data collection	Tissue and cell image were acquired by EVOS FL Auto software v1.7.<br>Heat-map analysis was performed using a PermutMatrix-1.9.3.<br>Microscopy image analysis was performed using ImageJ 1.51j8
Data analysis	GraphPad Prism 6 was used for data analysis<br>GSE data was analyzed by QIAGEN Ingeunity Pathway Analysis (I PA) software<br>Heat-map analysis was performed using a PermutMatarix-1.9.3<br>Microscopy image analysis was performed using ImageJ<br>KEGG pathway analysis was using GSEA 3.0

For manuscripts utilizing custom algorithms or software that are central to the research but not yet described in published literature, software must be made available to editors and reviewers. We strongly encourage code deposition in a community repository (e.g. GitHub). See the Nature Portfolio guidelines for submitting code & software for further information.

Data

Policy information about availability of data

All manuscripts must include a data availability statement. This statement should provide the following information, where applicable:

- Accession codes, unique identifiers, or web links for publicly available datasets
- A description of any restrictions on data availability
- For clinical datasets or third party data, please ensure that the statement adheres to our policy

Microarray data of Human OA chondrocytes (HYPERLINK "https://www.ncbi.nlm.nih.gov/geo/query/acc.cgi?acc=GSE16464" GSE16464, HYPERLINK "https://

www.ncbi.nlm.nih.gov/geo/query/acc.cgi?acc=GSE64394" GSE64394), primary mouse articular chondrocytes (IL-1b-treated; HYPERLINK "https://www.ncbi.nlm.nih.gov/geo/query/acc.cgi?acc=GSE104793" GSE104793, HIF-2a-overexpressed; HYPERLINK "https://www.ncbi.nlm.nih.gov/geo/query/acc.cgi?acc=GSE104794" GSE104794, ZIP8-overexpressed; HYPERLINK "https://www.ncbi.nlm.nih.gov/geo/query/acc.cgi?acc=GSE104795" GSE104795) for Fig.1g and OA-induced rat articular chondrocytes (HYPERLINK "https://www.ncbi.nlm.nih.gov/geo/query/acc.cgi?acc=GSE8077" GSE8077) for Fig. 3f were retrieved from GEO database. GSE data was analyzed by QIAGEN Ingeunity Pathway Analysis (IPA) software. Kyoto Encyclopedia of Genes and Genomes (KEGG) pathway analysis was performed by GSEA 3.0." Source data are provided with this paper.

Field-specific reporting

Please select the one below that is the best fit for your research. If you are not sure, read the appropriate sections before making your selection.

[x] Life sciences [ ] Behavioural & social sciences [ ] Ecological, evolutionary & environmental sciences

For a reference copy of the document with all sections, see nature.com/documents/nr-reporting-summary-flat.pdf

Life sciences study design

All studies must disclose on these points even when the disclosure is negative.

Sample size	For in vitro study, all experiments were performed at least three biologically independent experiments. For measuring positive area or intensity, 5-10 area/image were randomly selected in each experiments. No statistical method was used to determine sample size since this sample sizes are typical for the in vitro experiments. For in vivo study, groups of 4-7 mice and 5-7 littermates were used for each experiments which is sufficient to generate statistically significant results. The sample size for in vivo studies were chosen based on previous experience in the same experimental design.
Data exclusions	No data were excluded
Replication	All attempts for replication were successful by investigators of this study and confirmed by multiple investigators. For in vitro study, at least three biologically independent experiments were performed. Staining images were randomly captured and analyzed. For in vivo study, groups of 4-7 mice were used in experiments
Randomization	Wild type and knockout mice were randomly divided into each experimental groups. For iMACs culture, all of littermates were used in each experiments without selection. OA cartilage were collected from OA patients without secondary metabolic disease such as diabetes, cancers, rheumatic arthritis and other bone disease.
Blinding	For histological scoring and data collection, the investigators used sample ID and were not given grouping information. Staining image acquired randomly and assigned sample ID. Color thresholds for staining area measuring were equally applied to all images. Data analysis was performed and confirmed by multiple investigators.

Reporting for specific materials, systems and methods

We require information from authors about some types of materials, experimental systems and methods used in many studies. Here, indicate whether each material, system or method listed is relevant to your study. If you are not sure if a list item applies to your research, read the appropriate section before selecting a response.

Materials & experimental systems

n/a	Involved in the study	
[ ]	[x]	Antibodies
[ ]	[x]	Eukaryotic cell lines
[x]	[ ]	Palaeontology and archaeology
[ ]	[x]	Animals and other organisms
[ ]	[x]	Human research participants
[x]	[ ]	Clinical data
[x]	[ ]	Dual use research of concern

Methods

n/a	Involved in the study	
[x]	[ ]	ChIP-seq
[x]	[ ]	Flow cytometry
[x]	[ ]	MRI-based neuroimaging

Antibodies

Antibodies used	ACOT12: Mybiosource, #MBS273317, anti-Rabbit<br>GAPDH: Bioworld, #AP0066, Anti-Rabbit<br>ADAMTS4: Abcam, #Ab28285, Anti-Rabbit<br>FASN: Cell signaling, #3180, Anti-Rabbit<br>HA-tag: Cell singnaling, #3724, Anti-Rabbit<br>MMP13: BioVision, #3533, Anti-Rabbit

March 2021

	PPARA: Abcam, #Ab8934, Anti-Rabbit<br>SCD1: Abcam, #Ab19862, Anti-Mouse<br>HRP-conjugated rabbit secondary antibody: Bethyl laboratories, A120-101P, Goat anti-rabbit IgG
Validation	Most antibodies used in this study are commercial.<br>1. ACOT12, reactivity : human, mouse, rat and bovine, application : IHC-P, WB, manufacturer's website : https://www.mybiosource.com/polyclonal-bovine-human-mouse-ovine-rat-antibody/acot12/273137 (this product is discontinued)<br>2. GAPDH, reactivity : human, mouse and rat, application : WB, manufacturer's website : https://www.bioworlde.com/Primary-Antibodies/22934.html<br>3. ADAMTS4, reactivity : human, mouse* and monkey, application : IHC*, WB, manufacturer's website : https://www.abcam.com/adamts4-antibody-carboxyterminal-end-ab28285.html (This product is discontinued, reactivity and application were validated by published paper (*DOI : 10.1096/fj.201802132RR, 10.1111/acel.12665)<br>4. FASN, reactivity : human, mouse and rat, application : WB, IP, IHC and IF, manufacturer's website : https://www.cellsignal.com/products/primary-antibodies/fatty-acid-synthase-c20g5-rabbit-mab/3180<br>5. HA-tag, reactivity : All, application : WB, IP, IHC, IF and chIP, manufacturer's website : https://www.cellsignal.com/products/primary-antibodies/ha-tag-c29f4-rabbit-mab/3724<br>6. MMP13, reactivity : human, mouse, rat, horse and bovine, application : WB, IP, IHC, manufacturer's website : https://www.biovision.com/mmp-13-antibody.html<br>7. PPARA, reactivity : human, mouse and rat, application : ELISA, ICC/IF, IHC and WB, manufacturer's website : https://www.abcam.com/ppar-alpha-antibody-ab8934.html (This product is discontinued)<br>8. SCD1, reactivity : human, mouse* and rat, application : ICC, WB, IHC, IP and F, manufacturer's website : https://www.abcam.com/scd1-antibody-cde10-ab19862.html (reactivity was validated by published paper (*DOI : 10.1186/s12986-021-00570-3, 10.1186/s12986-020-00454-y)<br>9. HRP-conjugated rabbit secondary antibody manufacturer's website : https://www.bethyl.com/product/A120-101ADN"

Eukaryotic cell lines

Policy information about cell lines

Cell line source(s)	Normal human articular cartilage, OA articular cartilage
Authentication	Microscopic observation, Acian Blue staining, Type II collagen level
Mycoplasma contamination	Cell line was confirmed with no bacterial, fungi, mycoplasma, virus.
Commonly misidentified lines<br>(See ICLAC register)	*Name any commonly misidentified cell lines used in the study and provide a rationale for their use.*

Animals and other organisms

Policy information about studies involving animals; ARRIVE guidelines recommended for reporting animal research

Laboratory animals	8-weeks-old male and 5-days-old littermates C57BL/6N, Acot12-/- and Ppara-/- mice were used in this study. All mice housed at 23 ± 1خ with light/dark cycles and humedity of 50 ± 5% with food and water available ad libitum
Wild animals	None
Field-collected samples	None
Ethics oversight	All animal studies were approved by the Wonkwang University Animal Care and Use Committee (#WKU18-23, WKU19-09, WKU20-61) and were in compliance with the institutional guidelines.

Note that full information on the approval of the study protocol must also be provided in the manuscript.

Human research participants

Policy information about studies involving human research participants

Population characteristics	cartilages from OA patients undergo total knee replacement (TKR) surgery after being diagnosed with severe OA in University hospital were used. The study was performed with the cartilages of female since In our university, more than 90% of TKR patients are female with age between 60 to 85. We ruled out patients with secondary metabolic disease such as diabetes, cancers, rheumatic arthritis and other bone disease and patients with BMI≥30kg.
Recruitment	Patients whose scheduled for TKR surgery were recruited from University hospital with written informed consent. We ruled out patients with secondary metabolic disease such as diabetes, cancers, rheumatic arthritis and other bone disease and patients with BMI≥30kg between age of 62 to 75 to avoid the secondary effect from other disease, aging, obses etc besides from osteoarthritis.

Ethics oversight	Human cartilage tissue collection was approved by the Human Subjects Committee of Wonkwang University Hospital (WKUH 201605-HRBR-041) and studies were performed in compliance with the institutional guidelines. Written informed consent was obtained from all adult patients or at least one guardian of each patient prior to the start of the experiment.

Note that full information on the approval of the study protocol must also be provided in the manuscript.
